# Relative validity of the Chrono-Nutrition Behavior Questionnaire (CNBQ) against 11-day event-based ecological momentary assessment diaries of eating

**DOI:** 10.1186/s12966-025-01740-9

**Published:** 2025-04-25

**Authors:** Kentaro Murakami, Nana Shinozaki, Tracy A. McCaffrey, M. Barbara E. Livingstone, Shizuko Masayasu, Satoshi Sasaki

**Affiliations:** 1 Department of Social and Preventive Epidemiology, School of Public Health, University of Tokyo, Tokyo, 113 - 0033 Japan; 2 Department of Nutrition, Dietetics and Food, Monash University, Clayton, Melbourne, Australia; 3 Nutrition Innovation Centre for Food and Health (NICHE), School of Biomedical Sciences, Ulster University, Coleraine, UK; 4 Ikurien-Naka, Ibaraki, Japan

**Keywords:** Chrononutrition, Temporal eating patterns, Meal timing, Meal regularity, Eating window, Chronotype, Sleep, Questionnaire, Ecological momentary assessment, Epidemiology

## Abstract

**Background:**

A growing number of studies have investigated chrononutrition-related variables in relation to health outcomes. However, only a few questionnaires specifically designed for assessing chrononutrition-related parameters have been validated. We aimed to examine the relative validity of the Chrono-Nutrition Behavior Questionnaire (CNBQ) against 11-day event-based ecological momentary assessment (EMA) diaries of eating.

**Methods:**

Informed by previous research, we developed the CNBQ for the comprehensive assessment of chrononutrition-related parameters, including sleep variables, eating frequency, timing of eating, duration of eating occasions, duration of eating windows, and time interval between sleep and eating, for workdays and non-workdays separately. Between February and April 2023, a total of 1050 Japanese adults aged 20–69 years completed the online CNBQ and subsequently kept event-based EMA food diaries for 11 days, including 6.5 workdays and 4.5 non-workdays on average.

**Results:**

Mean differences between estimates derived from the CNBQ and the EMA food diaries were < 10% for most of the variables examined, both for workdays (27 of 33; 82%) and non-workdays (25 of 33; 76%), and for variables based on differences between workdays and non-workdays, such as eating jetlag (5 of 6; 83%). Spearman correlation coefficients between estimates based on the CNBQ and estimates based on the EMA food diaries were ≥ 0.50 for 26 variables (79%) on workdays and 22 variables (67%) on non-workdays (e.g., mid-sleep time; total eating frequency; timing of first eating occasion, last eating occasion, first meal, and last meal; duration of first meal and last meal; duration of eating window; eating midpoint; and time interval between wake time and first eating occasion and between last meal and sleep time), and 2 variables based on differences between workdays and non-workdays (e.g., eating jetlag base on breakfast timing). Bland–Altman analysis showed that the limits of agreement were wide and that the bias of overestimation by the CNBQ was proportional as mean estimates of the CNBQ and EMA food diaries increased.

**Conclusions:**

These findings suggest that the relative validity of the CNBQ justifies its use in estimating mean values and ranking individuals for the majority of chrononutrition-related parameters.

**Supplementary Information:**

The online version contains supplementary material available at 10.1186/s12966-025-01740-9.

## Background

Circadian rhythms—daily rhythms in our body that repeat every 24 h—are controlled by the master circadian clock in the suprachiasmatic nuclei of the hypothalamus and regulate daily sleep–wake rhythms, feeding behavior, and hormone secretion [1]. These circadian rhythms can be affected by entrainment factors (*zeitgebers*), such as light, hormones, and food intake [2]. Proper functioning of this system is essential for maintaining optimal metabolic health [3]. The typology of an individual’s circadian rhythm is summarized in the concept of chronotype, referring to an individual’s activity-rest preference over a 24-h period [4]. Individuals who preferentially wake up early and are active in the morning are said to be morning chronotypes, while those who preferentially wake up late and are night-oriented are said to be evening chronotypes [5]. Similarly, in later chronotypes (more evening-type), food consumption is temporally shifted to later in the day, whereby individuals eat later in the day and consume more energy toward the end of the day [6].

Recently, chrononutrition—the science that combines elements of nutritional research and chronobiology—has received increasing attention [7, 8] as a growing body of literature reveals a possible association between chronotype, temporal eating patterns, and health [2, 6–14]. For example, having a later first meal (later than 9 AM compared to earlier than 8 AM) and last meal of the day (later than 9 PM compared to earlier than 8 PM) was associated with a higher risk of cardiovascular disease in a prospective cohort study of French adults [15]. In the US, a prospective cohort study showed that, compared with men with a reported eating frequency of 3 times per day, those who reported an eating frequency of 1–2 times per day had a higher risk of type 2 diabetes [16]. Additionally, another US prospective cohort study showed a lower risk of 5-year incident obesity among women with stable breakfast consumption habits (i.e., those who always or never ate breakfast) compared to those with irregular breakfast habits (i.e., those who ate breakfast 3–4 days per week) [17]. Furthermore, several meta-analyses of randomized controlled trials have shown that time-restricted eating achieves weight loss similar to continuous energy restriction [18] and improves blood pressure, glucose, and lipid profiles [19, 20]. Moreover, a cross-sectional study in young Spanish adults showed a positive association of the eating midpoint difference between weekdays and weekend days (i.e., eating jetlag) with body mass index (BMI) [21]. Thus, chrononutrition encompasses a wide range of eating behaviors, including the clock time of the first and last food intake, the frequency and regularity of eating, time-restricted eating (or the duration of eating window), and eating jetlag [2, 8–11, 13, 14].

Nevertheless, chrononutrition research is hampered by the limited availability of standardized and validated tools that can be used in large-scale epidemiologic studies [11]. The appropriate modification of traditional tools to assess actual eating behaviors, such as 24-h dietary recall [13, 22] and food diary [15, 23], are viable options. However, these methods place a high burden on both participants and researchers [11], which is amplified by the need for multiple-day data collection to capture habitual eating patterns [24]. Conversely, traditional dietary assessment questionnaires, which are the mainstay of large-scale studies, are generally not designed to capture temporal patterns of eating [11, 25] and may accelerate the use of additional questions with uncertain validity regarding these variables [16, 17, 26–28]. As a result, the development of a single but comprehensive questionnaire to assess every aspect of chrononutrition-related behaviors is warranted [6, 11, 25, 29]. In nutritional epidemiology, it is best practice to evaluate the validity of a newly developed questionnaire before its use [30]. It is widely acknowledged that a food diary, a type of ecological momentary assessment (EMA), is the first method of choice for validating dietary questionnaires [30]. This is because in EMA, participants’ current eating behaviors are repeatedly sampled in their natural environment in real-time [31, 32], helping reduce recall bias, maximize ecological validity (i.e., generalizability to people’s daily lives and the natural environment), and capture intra-individual behaviors over time and across settings (e.g., workdays vs non-workdays) in a real-world context [31, 32].

To date, a small number of questionnaires on chrononutrition-related parameters have been specifically developed and validated [29, 33–37]. However, these questionnaires are limited in terms of their scope and relevance [2, 6, 11]. Specifically, they only assess meal timing and eating frequency [35–37], do not distinguish between workdays and non-workdays and, consequently, do not account for variables based on differences between workdays and non-workdays [29, 33–37], and do not assess chronotype [33–37]. Therefore, to our knowledge, no attempt has been made to validate a questionnaire for a comprehensive assessment of chrononutrition-related parameters, in comparison to an EMA tool. It is important to assess a comprehensive set of chrononutrition-related parameters, as the variables having the greatest pertinence to chrononutrition research have not yet been identified [6, 36]. To address these gaps, the aim of this study was to examine the relative validity of the newly developed Chrono-Nutrition Behavior Questionnaire (CNBQ) against 11-day event-based EMA diaries of eating in a large sample of Japanese adults.

## Methods

### Overview of the study procedure

**Fig. 1:**
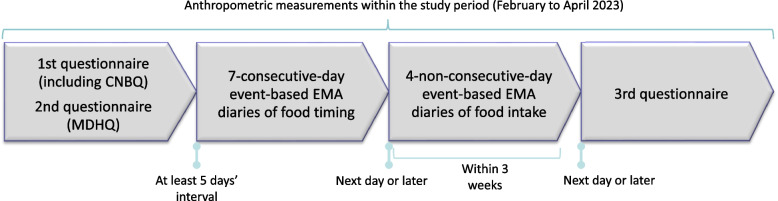
Schedule of the 5 W study. 5 W study, Who, What, When, Where, and Why for Healthy Eating study; CNBQ, Chrono-Nutrition Behavior Questionnaire; MDHQ, Meal-based Diet History Questionnaire; EMA, ecological momentary assessment. In the present analysis, data derived from the 2nd and 3rd questionnaires were not used

This analysis was based on data from the Who, What, When, Where, and Why for Healthy Eating study (hereafter referred to as 5 W study). Using a cross-sectional design, the 5 W study was conducted between February and April 2023. The study schedule is shown in Fig. 1. First, each participant was asked to answer two web-based questionnaires (using the Google Forms platform), including the CNBQ and the Meal-based Diet History Questionnaire (MDHQ) [38–41]. Then, 7-consecutive-day event-based EMA diaries of food timing and 4-non-consecutive-day event-based EMA diaries of food intake (i.e., weighed dietary record) were conducted. The final component was the 3rd web-based questionnaire. Information from the 2nd (i.e., MDHQ) and 3rd questionnaires was not used in the present analysis. During the study period, anthropometric measurement was also conducted. Each participant received an honorarium to the value of 4000 Japanese yen (US$25.20 as of 19 June 2024) after completing the study. The study was conducted in accordance with the guidelines of the Declaration of Helsinki, and all procedures were approved by the Ethics Committee of the University of Tokyo Faculty of Medicine (protocol code: 2022235 NI; date of approval: 24 November 2022). Written informed consent was obtained from all participants.

### Study participants

The target population comprised apparently healthy Japanese aged 20–69 years living in private households in Japan. Initially, 26 (of 47) prefectures, which cover > 72% of the total population of Japan, were selected on the basis of geographical diversity and feasibility of the survey. A total of 264 research dietitians with expertise in collecting dietary record data [23, 41] agreed to support the study and were responsible for recruitment of participants and data collection. On the basis of the availability of research dietitians and financial resources (assuming 4–5 participants per research dietitian), we decided to include 110 individuals for each of sex-specific, five 10-year age categories (i.e., 20–29, 30–39, 40–49, 50–59, and 60–69 years), resulting in 1110 individuals in total. For recruitment, we aggregated the 26 prefectures into 7 regions. The target number of participants for each region (*n* = 100–200) was determined mainly based on the number of research dietitians available. To minimize the dropout rate, potential participants were restricted to individuals who had a full understanding of the study’s protocol and agreed to complete the entire survey procedure. Exclusion criteria were dietitians, individuals living with a dietitian, those who were currently receiving dietary counselling from a doctor or dietitian, those taking insulin treatment for diabetes, those undergoing dialysis treatment, those without sufficient internet access, those who had difficulty answering the web-based questionnaires, and pregnant or lactating women. We did not exclude night shift workers but asked them not to conduct the 4-non-consecutive-day EMA diaries of food intake on night shift working days, or on the days immediately preceding and following these days. Participation of only one person per household was permitted. Using the snowball sampling procedure, the number of individuals approached for this study was 1796, 1110 of whom agreed to participate (response rate 61.8%).

### Chrono-Nutrition Behavior Questionnaire

Development of the CNBQ was informed by existing questionnaires [29, 33–37] and several reviews [6, 11, 12] in the field of temporal patterns of eating and chrononutrition. We designed the CNBQ with the aim of comprehensively assessing chrononutrition-related parameters. Supplemental Tables 1 and 2 show the original Japanese and translated English versions of CNBQ, respectively. An in-house pretest was conducted with staff and students from the Department of Social and Preventive Epidemiology, School of Public Health, University of Tokyo, after which some modifications were made. The CNBQ consisted of three parts. Part 1 included general questions on engagement in shift work and the number of paid work (or school) days per week. For participants without a paid job or school (e.g., primary homemakers and caregivers), we asked them to consider the days when their partner was engaged in paid work as workdays and the days their partner was not as non-workdays. If the partner of a participant without a paid job (or school) did not have a paid job or if the participants did not have a partner, we asked them to consider weekdays (Mondays to Fridays) as workdays and weekend days (Saturdays and Sundays) as non-workdays.

Participants were then asked about sleep and chrononutrition behaviors on workdays (in Part 2) and non-workdays (in Part 3) separately. Question items included sleep habits (sleep time, wake time, and use of alarm clock), based on the concept of Munich ChronoType Questionnaire [42, 43]; timing of eating; and duration of eating occasions. For the timing of eating and duration of eating occasions, we provided six pre-specified eating occasion slots (i.e., breakfast, morning snack, lunch, afternoon snack, dinner, and evening snack), since our previous analysis based on 8-day weighed food records collected over a single year (2 days in each season) from a large sample of general Japanese (*n* = 4032) showed clear peaks in the timing of these eating occasions [23]. Also, as snack frequencies are generally low in Japanese [23], we asked participants to combine their snacking events into one within the eating occasion slots (by averaging the timing and summing up the time spent eating).

In the CNBQ, participants were asked to only consider eating occasions which consisted of at least one food but not consider those which consisted of beverages or water only. Participants who answered that they worked seven days a week (in Part 1) were not provided with a series of questions on non-workdays (i.e., Part 3). Reference time period in the CNBQ was defined as the preceding month (to correspond with the time frame of the MDHQ [38]).

### Eleven-day event-based ecological momentary assessment diaries of eating

As reference method in this study, we selected 11-day event-based EMA food diaries, namely a combination of 7-consecutive-day food timing diaries and 4-non-consecutive-day (2 workdays and 2 non-workdays) weighed food intake diaries. Using this combination, we expected to collect data on a sufficient number of non-workdays (at least 4 days) while not compromising the feasibility and simplicity of the conduct of the survey. After receiving written and verbal instructions by a research dietitian, as well as an example of a completed food timing diary sheet, each participant was requested to maintain a record of food timing (start and finish clock times), both in and out of the home. Participants were also asked to select the most appropriate eating occasion name from the prescribed list (breakfast, lunch, dinner, and snack), as well as to indicate if the recording day was either a workday or a non-workday. The definitions of workdays and non-workdays were identical to those used in the CNBQ. To minimize the burden on participants, they were not asked to provide information about the foods they consumed, nor were they asked to report the occasions on which they consumed only beverages or water. Conversely, participants were also asked to record the time of going to bed, time of finishing preparation for sleep, and sleep latency (i.e., the length of time of the transition from full wakefulness to sleep) on the previous day and the time of waking. Research dietitians checked the completeness of the food timing diaries via phone, internet, or in-person three times during the 7-day period (for day 1, days 2–3, and days 4–7), and if necessary, additional information was added. The procedure used to complete the food intake diaries which has been described thoroughly elsewhere [23, 41] was similar to the food timing diaries, but more detailed descriptions were requested, including the name and amount of each food and beverage consumed. The food intake diary sheets were submitted directly to the research dietitian after the completion of entries for each day, who then reviewed the sheets and, whenever necessary, sought additional information or modified the sheets via phone or in-person interview.

### Creation of chrononutrition-related parameters

All responses to the web-based questionnaire (including CNBQ) were automatically entered into a spreadsheet format downloaded from Google Drive. Data were minimally cleaned (e.g., times that logically should be pm rather than am were adjusted) to avoid overestimating the validity of the test method (CNBQ). All collected EMA food diaries were thoroughly reviewed by the research dietitians and trained staff at the study center, and any ambiguity was solved by asking the research dietitians in charge. All information from the EMA food diaries was then manually entered into a spreadsheet by trained staff at the study center in duplicate, and any discrepancies were checked and corrected. All eating occasions recorded in food intake diaries which consisted of beverages or water only were excluded from this analysis.

For each of the CNBQ and EMA food diaries, we created sleep variables and temporal patterns of eating variables for workdays and non-workdays separately. Detailed descriptions of these variables can be found in Supplemental Table 3. In short, four sleep variables were considered, namely sleep time, wake time, sleep duration, and mid-sleep time [29]. Temporal patterns of eating variables included three eating frequency variables (meal frequency, snack frequency, and total eating frequency) [23, 29, 37]; nine variables of start time of eating (first eating occasion, last eating occasion, first meal, last meal, first snack, last snack, breakfast, lunch, and dinner) [23, 29, 33, 37]; nine variables of duration of eating occasions (same as the start time of eating) [23]; four eating window variables (duration of eating window and eating midpoint (i.e., midpoint of clock time of eating window) based on the start time of first eating occasion and the start time of last eating occasion, and those based on the start time of first eating occasion and the finish time of last eating occasion (i.e., start time of last eating occasion plus time spent on last eating occasion)) [11, 29, 33, 34, 44]; and four variables of time interval between sleep and eating (wake time and first eating occasion, wake time and first meal, last eating occasion and sleep time, and last meal and sleep time) [11, 29, 33, 34, 45]. In this study, we considered that meals included breakfast, lunch, and dinner while snacks included all other eating occasions, including morning snack, afternoon snack, and evening snack [23, 46].

﻿Additionally, we created several variables based on workday and non-workday differences. Specifically, social jetlag was calculated as the absolute difference between mid-sleep time on workdays and non-workdays [47]. Also, eating jetlag based on eating window, breakfast timing, lunch timing, and dinner timing was calculated as the absolute difference between workdays and non-workdays in each variable [21]. Furthermore, chronotype was defined based on the Munich ChronoType Questionnaire concept of chronotype [42], as follows.

- For people whose sleep duration on non-workdays was longer than that on workdays, chronotype was defined as the midpoint of sleep on non-workdays, adjusted for possible sleep debt accumulated on workday nights, namely by subtracting half of the difference between sleep duration on non-workdays and sleep duration on workdays.
- For people whose sleep duration on non-workdays was equal to or shorter than that on workdays, chronotype was defined as the midpoint of sleep on non-workdays.

The calculation of chronotype was conducted for the entire sample, but also only for participants who do not use an alarm clock on non-workdays, as suggested by the original authors [42].

### Assessment of other variables

In this study, biological sex was self-selected as either male or female. Age at the start of the study was calculated based on birth date. Based on BMI (in kg/m^2^) calculated using body weight and height, which in many cases were measured by either a family member or research dietitian, four categories of weight status were created: underweight (< 18.5), normal weight (≥ 18.5 to < 25), overweight (≥ 25 to < 30), and obese (≥ 30) [48]. Self-reported information on the following variables was also used in this study (categorization shown in parentheses): smoking status (never, past, and current), living alone (yes or no), education level (junior high school or high school, junior college or technical school, university or higher, and other), annual household income (< 4 million Japanese yen, ≥ 4 to < 7 million Japanese yen, ≥ 7 million Japanese yen, and unknown/do not want to answer), and employment status (none, student, part-time paid job, and full-time paid job).

### Analytic sample

**Fig. 2:**
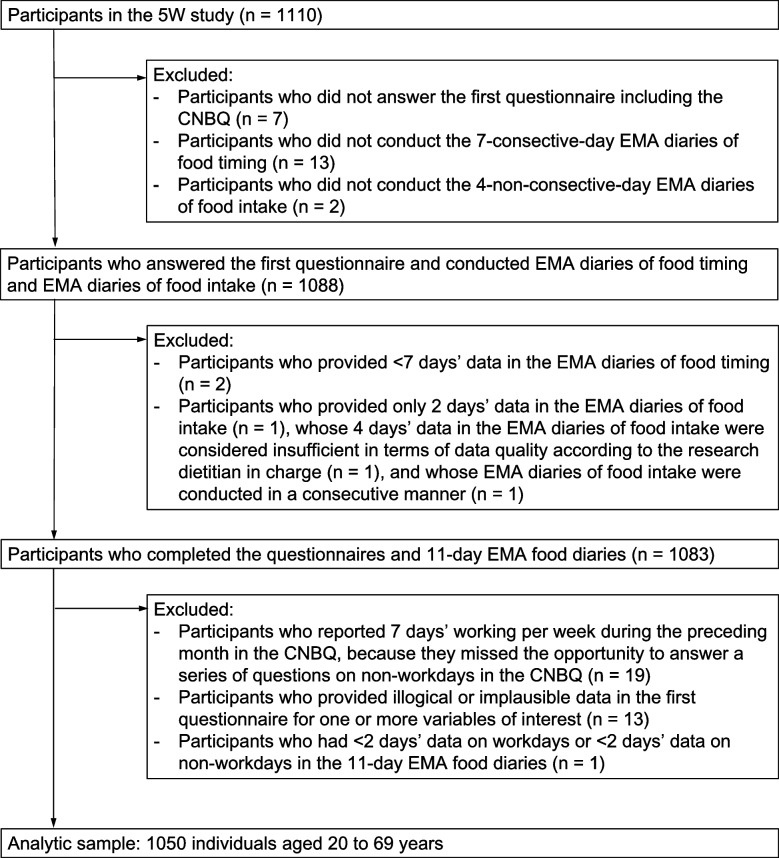
Flow diagram of participant selection in the present analysis. 5 W study, Who, What, When, Where, and Why for Healthy Eating study; CNBQ, Chrono-Nutrition Behavior Questionnaire; EMA, ecological momentary assessment

Figure 2 shows a flow diagram of participant selection in the present analysis. From the initial sample of 1110 participants, we retained participants who answered the first questionnaire and conducted EMA diaries of food timing and EMA diaries of food intake (*n* = 1088). We then excluded participants who provided < 7 days’ data in the EMA diaries of food timing (*n* = 2) or only 2 days’ data in the EMA diaries of food intake (*n* = 1), those whose 4 days’ data in the EMA diaries of food intake were considered insufficient in terms of data quality according to the research dietitian in charge (*n* = 1), and those whose EMA diaries of food intake were conducted in a consecutive manner (*n* = 1). We finally excluded participants who reported 7 days’ working per week during the preceding month in the CNBQ (because they missed the opportunity to answer a series of questions on non-workdays in the CNBQ; *n* = 19), provided illogical or implausible data in the first questionnaire for one or more variables of interest (*n* = 13), or had < 2 days’ data on workdays or < 2 days’ data on non-workdays in the 11-day EMA food diaries (*n* = 1). Thus, the final analysis sample comprised 1050 participants.

### Statistical analysis

Statistical analyses were performed using the SAS statistical software (version 9.4; SAS Institute Inc.). All reported *P* values are 2-tailed, and *P* values < 0.05 were considered statistically significant. Data on sample characteristics are presented as means and standard deviations (SDs) for continuous variables and as the numbers and percentages of participants for categorical variables. All chrononutrition-related parameters were expressed using means and SDs. All analyses were conducted for workday data and non-workday data separately, except for variables based on differences between workdays and non-workdays (e.g., social jetlag). To assess estimation ability at the group level, mean values of estimates derived from the CNBQ were compared with those derived from the EMA food diaries using the paired t-test. In accordance with a previous review [49], we considered the group-level estimation ability good when *P* > 0.05; furthermore, given the large sample size, a group-level bias of < 10% was classified as acceptable (i.e., indication of a relatively similar mean intake) [49]. The use of nonparametric testing (Wilcoxon signed-rank test) and median values did not change the results materially (data not shown). In addition, to assess agreement, mean differences (with 95% limits of agreement) between the CNBQ-based estimates and EMA food diary-based estimates were calculated using Bland–Altman analysis [50]; for selected variables, we also displayed the Bland–Altman plots [50], accompanied by examination of proportional bias using the linear regression analysis [51]. Further, the ability of the CNBQ to rank individuals in a population was assessed using Spearman correlation coefficients between the CNBQ-based estimates and EMA food diary-based estimates. Correlations in nutritional epidemiology validation studies tend to be on the order of 0.4–0.7 [52, 53]. Although there is no certain cutoff, low correlation values (e.g., < 0.4) were considered undesirable [54, 55]. In accordance with a previous validation study on the same topic [37], we considered Spearman correlation coefficients of 0.5 to be acceptable. The same analyses were repeated for male and female participants separately; for younger and older participants (divided by median age of 44 years in the present sample) separately; and for shift working participants (individuals who reported engagement in shift working during the preceding three months as assessed by the CNBQ) and non-shift working participants separately. These subgroup analyses were intended to provide insight into future studies based on the CNBQ, in which, for example, only female participants, younger participants, or non-shift working participants may be included.

## Results

This analysis included a total of 1050 Japanese adults aged 20–69 years who completed the CNBQ and subsequently kept event-based EMA food diaries for 11 days, including 6.5 workdays and 4.5 non-workdays on average (Table 1).

**Table 1:** Basic characteristics of study participants (*n* = 1050) ^a^

Variable	Value
Female sex [n (%)]	518 (49.3)
Age (years)	44.3 ± 13.9
BMI (kg/m^2^)	23.0 ± 3.9
Weight status [n (%)]	
Underweight (BMI < 18.5 kg/m^2^)	82 (7.8)
Normal weight (BMI ≥ 18.5 to < 25 kg/m^2^)	700 (66.7)
Overweight (BMI ≥ 25 to < 30 kg/m^2^)	204 (19.4)
Obese (BMI ≥ 30 kg/m^2^)	64 (6.1)
Smoking status [n (%)]	
Never	680 (64.8)
Past	188 (17.9)
Current	182 (17.3)
Living alone [n (%)]	173 (16.5)
Education level [n (%)]	
Junior high school or high school	274 (26.1)
Junior college or technical school	354 (33.7)
University or higher	419 (39.9)
Other	3 (0.3)
Annual household income [n (%)]	
< 4 million Japanese yen	294 (28.0)
≥ 4 to < 7 million Japanese yen	349 (33.2)
≥ 7 million Japanese yen	375 (35.7)
Unknown/do not want to answer	32 (3.1)
Employment status [n (%)]	
None	63 (6.0)
Student	25 (2.4)
Part-time paid job	159 (15.1)
Full-time paid job	803 (76.5)
Number of workdays during the preceding month (per week)	4.9 ± 0.8
Number of workdays during the 11-day EMA food diaries ^b^	6.5 ± 1.1
Number of non-workdays during the 11-day EMA food diaries ^b^	4.5 ± 1.1
Experience of shift work during the preceding three months [n (%)]	246 (23.4)
Night shift work during the period of 11-day EMA food diaries [n (%)] ^b^	55 (5.2)
Use of an alarm clock on workdays [n (%)]	897 (85.4)
Use of an alarm clock on non-workdays [n (%)]	391 (37.2)

*BMI* body mass index, *EMA* ecological momentary assessment

^a^Values are means ± standard deviations unless otherwise indicated

^b^The11-day EMA food diaries consisting of 7-consecutive-day food timing diaries and 4-non-consecutive-day food intake diaries

### Sleep and temporal patterns of eating variables on workdays

Table 2 shows sleep and temporal patterns of eating variables on workdays. Note that, to facilitate statistical analysis, all time-related variables in this and subsequent tables are shown in decimal format. For 12 of the 33 variables listed here, mean values of the CNBQ-based estimates did not significantly differ from the corresponding values of the EMA food diaries. For the remaining 21 variables, significant differences between the 2 instruments were observed. However, the magnitude of differences was relatively small (i.e., ≤ 7%), except for the 6 following variables: daily snack frequency (21.6% overestimation by CNBQ), duration of first eating occasion (23.6% overestimation), duration of last meal (11.9% underestimation), duration of first snack (12.1% underestimation), duration of dinner (12.0% underestimation), and time interval between last eating occasion and sleep time (11.3% underestimation). The Spearman correlation coefficients ranged from 0.28 (duration of last eating occasion) to 0.80 (start time of first eating occasion) with a median of 0.63 (25 th percentile 0.53; 75 th percentile 0.73). When separate analyses were conducted by sex (Supplemental Tables 4 and 5), age (Supplemental Tables 6 and 7), and shift working status (Supplemental Tables 8 and 9), the CNBQ showed a somewhat better ability in male participants, older participants, and non-shift working participants compared with their counterparts. For example, the median Spearman correlation coefficients were 0.65 in male participants compared with 0.59 in female participants; 0.64 in older participants compared with 0.60 in younger participants; and 0.68 in non-shift working participants compared with 0.52 in shift-working participants.

**Table 2:** Sleep and temporal patterns of eating variables on workdays ^a^

	CNBQ		EMA food diaries ^b^		Paired	Mean	Limit of	Spearman
	n	Mean (SD)	n	Mean (SD)	n	difference ^c^	Agreement ^d^	correlation ^e^
Sleep variables								
Sleep time (clock time; decimal)	1050	23.51 (1.58)	1050	23.56 (1.38)	1050	− 0.05	− 3.16, 3.06	0.71
Wake time (clock time; decimal)	1050	6.24 (1.17)	1050	6.38 (1.22)	1050	− 0.14****	− 1.75, 1.47	0.79
Daily sleep duration (hours; decimal)	1050	6.66 (1.16)	1050	6.53 (1.03)	1050	0.13****	− 1.70, 1.96	0.63
Mid-sleep time (clock time; decimal)	1050	3.02 (1.77)	1050	3.24 (1.45)	1050	− 0.21****	− 2.82, 2.40	0.79
Daily eating frequency (number)								
Meals	1050	2.78 (0.45)	1050	2.77 (0.39)	1050	0.01	− 0.56, 0.58	0.69
Snacks	1050	1.20 (0.98)	1050	0.99 (0.90)	1050	0.21****	− 1.72, 2.14	0.50
Total	1050	3.99 (1.13)	1050	3.76 (1.02)	1050	0.22****	− 1.82, 2.26	0.55
Start time of eating (clock time; decimal)								
First eating occasion	1050	8.06 (2.44)	1050	8.07 (2.17)	1050	− 0.01	− 2.90, 2.88	0.80
Last eating occasion	1050	20.37 (1.72)	1050	20.09 (1.24)	1050	0.27****	− 2.58, 3.12	0.58
First meal	1050	8.14 (2.61)	1050	8.23 (2.30)	1050	− 0.09	− 2.97, 2.79	0.80
Last meal	1050	19.40 (1.34)	1050	19.53 (1.17)	1050	− 0.13****	− 2.24, 1.98	0.70
First snack	755	15.50 (4.38)	907	15.67 (3.63)	710	− 0.17	− 8.30, 7.96	0.47
Last snack	755	19.35 (3.35)	907	17.99 (3.02)	710	1.28****	− 5.40, 7.96	0.39
Breakfast	857	7.04 (0.89)	972	7.27 (1.00)	852	− 0.11****	− 1.34, 1.12	0.77
Lunch	1025	12.44 (0.81)	1045	12.59 (0.73)	1024	− 0.13****	− 1.49, 1.23	0.61
Dinner	1040	19.47 (1.15)	1049	19.64 (1.07)	1040	− 0.17****	− 1.75, 1.41	0.74
Duration of eating occasion (minutes; decimal)								
First eating occasion	1050	14.25 (7.38)	1050	11.53 (4.99)	1050	2.72****	− 11.80, 17.24	0.45
Last eating occasion	1050	17.47 (18.89)	1050	18.31 (9.30)	1050	− 0.84	− 39.52, 37.84	0.28
First meal	1050	14.25 (7.38)	1050	14.51 (7.14)	1050	− 0.26	− 13.65, 13.13	0.58
Last meal	1050	26.86 (16.21)	1050	30.47 (16.67)	1050	− 3.61****	− 32.88, 25.66	0.58
First snack	736	9.90 (11.94)	907	11.95 (18.28)	695	− 1.44*	− 32.25, 29.37	0.40
Last snack	736	12.40 (15.60)	907	13.02 (19.28)	695	− 0.22	− 34.78, 34.34	0.43
Breakfast	861	13.50 (6.14)	972	13.07 (6.29)	856	0.18	− 10.99, 11.35	0.60
Lunch	1028	17.38 (7.24)	1045	18.20 (7.41)	1027	− 0.72***	− 13.20, 11.76	0.62
Dinner	1039	26.84 (15.94)	1049	30.48 (16.69)	1039	− 3.64****	− 32.51, 25.23	0.58
Eating window								
Duration of eating window 1 (hours; decimal)^f^	1050	12.31 (2.78)	1050	12.03 (2.32)	1050	0.28****	− 3.75, 4.31	0.66
Duration of eating window 2 (hours; decimal)^g^	1050	12.60 (2.76)	1050	12.33 (2.32)	1050	0.27****	− 3.82, 4.36	0.64
Eating midpoint 1 (clock time; decimal)^f^	1050	14.21 (1.59)	1050	14.08 (1.34)	1050	0.13****	− 1.91, 2.17	0.74
Eating midpoint 2 (clock time; decimal)^g^	1050	14.36 (1.61)	1050	14.23 (1.36)	1050	0.12***	− 1.95, 2.19	0.73
Time interval between sleep and eating (hours; decimal)								
Wake time and first eating occasion	1050	1.84 (2.32)	1050	1.77 (1.95)	1050	0.07	− 2.77, 2.91	0.73
Wake time and first meal	1050	1.92 (2.46)	1050	1.90 (2.10)	1050	0.03	− 2.86, 2.92	0.73
Last eating occasion and sleep time	1050	2.95 (1.59)	1050	3.33 (1.21)	1050	− 0.38****	− 3.39, 2.63	0.45
Last meal and sleep time	1050	3.72 (1.58)	1050	3.67 (1.32)	1050	0.05	− 2.53, 2.63	0.66

*CNBQ* Chrono-Nutrition Behavior Questionnaire, *EMA* ecological momentary assessment, *SD* standard deviation

^a^ Meals included breakfast, lunch, and dinner. All time-related variables are shown in decimal format, with the unit of *clock time; decimal* (e.g., 23.51 means 11:30 PM, while 6.24 means 6:14 AM); *hours; decimal* (e.g., 6.66 means a duration of 6 h 40 min); or *minutes; decimal* (e.g., 14.25 means a duration of 14 min 15 s)

^b^ Based on 2–9 days’ data (median 7 days)

^c^ Calculated as the CNBQ-based value minus the EMA food diary-based value (at the individual level). Paired comparison was made using the paired t-test: * *P* < 0.05, ** *P* < 0.01, *** *P* < 0.001, and **** *P* < 0.0001

^d^ Calculated as mean difference plus-minus 1.96 SD of the difference

^e^ All values were significant (*P* < 0.0001)

^f^ Calculated using the start time of first eating occasion and the start time of last eating occasion

^g^ Calculated using the start time of first eating occasion and the finish time of last eating occasion

### Sleep and temporal patterns of eating variables on non-workdays

Table 3 shows sleep and temporal patterns of eating variables on non-workdays. For nine variables, no significant differences were observed between mean values of the CNBQ-based and EMA food diary-based estimates. Although there were significant differences in the other 24 variables, the magnitude of differences was relatively small (i.e., ≤ 8%), except for daily snack frequency (19.3% overestimation by CNBQ), duration of first eating occasion (20.5% overestimation), duration of last meal (16.9% underestimation), duration of first snack (13.2% underestimation), duration of last snack (13.5% underestimation), duration of lunch (10.3% underestimation), duration of dinner (16.4% underestimation), and time interval between last eating occasion and sleep time (11.6% underestimation). The Spearman correlation coefficients ranged from 0.20 (duration of last eating occasion) to 0.77 (mid-sleep time) with a median of 0.54 (25 th percentile 0.47; 75 th percentile 0.65). Results of separate analyses by sex (Supplemental Tables 10 and 11), age (Supplemental Tables 12 and 13), and shift-working status (Supplemental Tables 14 and 15) were similar.

**Table 3:** Sleep and temporal patterns of eating variables on non-workdays ^a^

	CNBQ		EMA food diaries ^b^		Paired	Mean	Limit of	Spearman
	n	Mean (SD)	n	Mean (SD)	n	difference ^c^	Agreement ^d^	correlation ^e^
Sleep variables								
Sleep time (clock time; decimal)	1050	23.62 (2.61)	1050	23.94 (1.67)	1050	− 0.32***	− 5.54, 4.90	0.65
Wake time (clock time; decimal)	1050	7.46 (1.59)	1050	7.36 (1.50)	1050	0.10**	− 2.15, 2.35	0.74
Daily sleep duration (hours; decimal)	1050	7.63 (1.42)	1050	7.17 (1.24)	1050	0.46****	− 2.27, 3.19	0.47
Mid-sleep time (clock time; decimal)	1050	3.69 (1.59)	1050	3.82 (1.39)	1050	− 0.13**	− 2.78, 2.52	0.77
Daily eating frequency (number)								
Meals	1050	2.72 (0.49)	1050	2.69 (0.42)	1050	0.03*	− 0.74, 0.80	0.59
Snacks	1050	1.26 (1.01)	1050	1.06 (0.82)	1050	0.20****	− 1.74, 2.14	0.47
Total	1050	3.98 (1.17)	1050	3.75 (0.99)	1050	0.23****	− 1.91, 2.37	0.51
Start time of eating (clock time; decimal)								
First eating occasion	1050	9.13 (2.20)	1050	9.07 (1.97)	1050	0.06	− 2.90, 3.02	0.76
Last eating occasion	1050	19.81 (1.62)	1050	19.61 (1.19)	1050	0.20****	− 2.68, 3.08	0.51
First meal	1050	9.25 (2.40)	1050	9.19 (2.06)	1050	0.06	− 3.11, 3.23	0.76
Last meal	1050	18.81 (1.02)	1050	18.98 (1.02)	1050	− 0.17****	− 2.04, 1.70	0.61
First snack	752	14.57 (3.74)	949	14.87 (2.95)	726	− 0.23	− 7.74, 7.28	0.35
Last snack	752	18.80 (3.18)	949	17.26 (2.78)	726	1.43****	− 5.26, 8.12	0.35
Breakfast	803	8.13 (1.12)	961	8.28 (1.05)	796	0.00	− 1.69, 1.69	0.71
Lunch	1016	12.55 (0.77)	1040	12.74 (0.72)	1010	− 0.19****	− 1.71, 1.33	0.49
Dinner	1041	18.85 (0.89)	1048	19.14 (0.86)	1041	− 0.29****	− 1.70, 1.12	0.67
Duration of eating occasion (minutes; decimal)								
First eating occasion	1050	18.13 (10.38)	1050	15.05 (7.33)	1050	3.08****	− 18.01, 24.17	0.40
Last eating occasion	1050	19.90 (20.54)	1050	20.61 (11.73)	1050	− 0.71	− 44.55, 43.13	0.20
First meal	1050	18.13 (10.38)	1050	19.49 (12.98)	1050	− 1.37***	− 26.19, 23.45	0.54
Last meal	1050	29.99 (18.70)	1050	36.08 (21.65)	1050	− 6.08****	− 41.59, 29.43	0.55
First snack	751	12.41 (11.01)	949	14.34 (16.60)	726	− 1.89**	− 32.27, 28.49	0.32
Last snack	751	14.35 (15.50)	949	16.32 (21.46)	726	− 2.20**	− 37.80, 33.40	0.40
Breakfast	814	16.37 (7.51)	961	17.01 (8.53)	803	− 1.00***	− 16.14, 14.14	0.57
Lunch	1019	21.36 (10.29)	1040	23.88 (11.21)	1012	− 2.45****	− 24.60, 19.70	0.47
Dinner	1043	29.96 (18.46)	1048	35.80 (21.07)	1042	− 5.88****	− 40.25, 28.49	0.55
Eating window								
Duration of eating window 1 (hours; decimal)^f^	1050	10.68 (2.53)	1050	10.54 (2.06)	1050	0.13*	− 4.15, 4.41	0.54
Duration of eating window 2 (hours; decimal)^g^	1050	11.01 (2.48)	1050	10.89 (2.07)	1050	0.12	− 4.20, 4.44	0.52
Eating midpoint 1 (clock time; decimal)^f^	1050	14.47 (1.46)	1050	14.34 (1.26)	1050	0.13****	− 1.85, 2.11	0.74
Eating midpoint 2 (clock time; decimal)^g^	1050	14.63 (1.48)	1050	14.51 (1.29)	1050	0.12****	− 1.89, 2.13	0.74
Time interval between sleep and eating (hours; decimal)								
Wake time and first eating occasion	1050	1.68 (1.75)	1050	1.73 (1.41)	1050	− 0.05	− 3.03, 2.93	0.54
Wake time and first meal	1050	1.80 (1.92)	1050	1.84 (1.53)	1050	− 0.04	− 3.25, 3.17	0.57
Last eating occasion and sleep time	1050	3.66 (1.67)	1050	4.14 (1.34)	1050	− 0.48****	− 3.81, 2.85	0.39
Last meal and sleep time	1050	4.48 (1.55)	1050	4.50 (1.41)	1050	− 0.02	− 2.75, 2.71	0.59

*CNBQ* Chrono-Nutrition Behavior Questionnaire, *EMA* ecological momentary assessment, *SD* standard deviation

^a^ Meals included breakfast, lunch, and dinner. All time-related variables are shown in decimal format, with the unit of *clock time; decimal* (e.g., 23.62 means 11:37 PM, while 7.46 means 7:28 AM); *hours; decimal* (e.g., 7.63 means a duration of 7 h 38 min); or *minutes; decimal* (e.g., 18.13 means a duration of 18 min 8 s)

^b^ Based on 2–9 days’ data (median 4 days)

^c^ Calculated as the CNBQ-based value minus the EMA food diary-based value (at the individual level). Paired comparison was made using the paired t-test: * *P* < 0.05, ** *P* < 0.01, *** *P* < 0.001, and **** *P* < 0.0001

^d^ Calculated as mean difference plus-minus 1.96 SD of the difference

^e^ All values were significant (*P* < 0.0001)

^f^ Calculated using the start time of first eating occasion and the start time of last eating occasion

^g^ Calculated using the start time of first eating occasion and the finish time of last eating occasion

### Workday and non-workday differences variables

Social and eating jetlag variables and chronotype are shown in Table 4. The magnitude of mean differences between the CNBQ-based and EMA food diary-based estimates was relatively small (i.e., ≤ 9%) for all variables (except for eating jetlag based on eating midpoint 1, which showed 15.6% overestimation by CNBQ), although some of these reached statistical significance. The Spearman correlation coefficients ranged from 0.30 (eating jetlag based on lunch timing) to 0.71 (chronotype). Separate analyses by sex and age provided similar results, whereas the CNBQ showed somewhat better ability in non-shift working participants compared with shift-working participants (Supplemental Table 16); for example, the Spearman correlation coefficients ranged from 0.30 to 0.76 in non-shift working participants and from 0.23 to 0.58 in shift-working participants.

**Table 4:** Social and eating jetlag variables and chronotype ^a^

	CNBQ		EMA food diaries ^b^		Paired	Mean	Limit of	Spearman
	n	Mean (SD)	n	Mean (SD)	n	difference ^c^	Agreement ^d^	correlation ^e^
Social jetlag (hours; decimal)	1050	0.92 (1.63)	1050	0.96 (1.18)	1050	− 0.04	− 2.68, 2.60	0.41
Eating jetlag based on eating midpoint 1 (hours;decimal) ^f^	1050	0.89 (1.01)	1050	0.77 (0.73)	1050	0.12****	− 1.74, 1.99	0.34
Eating jetlag based on breakfast timing (hours;decimal)	759	1.17 (1.04)	916	1.14 (0.91)	753	0.10**	− 1.73, 1.92	0.57
Eating jetlag based on lunch timing (hours; decimal)	994	0.67 (0.73)	1036	0.64 (0.58)	989	0.05*	− 1.36, 1.46	0.30
Eating jetlag based on dinner timing (hours; decimal)	1033	0.80 (0.89)	1048	0.77 (0.74)	1033	0.04	− 1.55, 1.62	0.39
Chronotype (clock time; decimal)	1050	3.31 (1.54)	1050	3.53 (1.33)	1050	− 0.22****	− 2.97, 2.52	0.71
Chronotype (clock time; decimal) among non- users of clock alarm on non-workdays	659	3.31 (1.28)	659	3.59 (1.36)	659	− 0.28****	− 2.39, 1.83	0.71

*CNBQ* Chrono-Nutrition Behavior Questionnaire, *EMA* ecological momentary assessment, *SD* standard deviation

^a^ Meals included breakfast, lunch, and dinner. All variables are shown in decimal format, with the unit of *hours; decimal* (e.g., 0.92 means a duration of 0 h 55 min) or *clock time; decimal* (e.g., 3.31 means 3:19 AM)

^b^ Based on 11 days’ data: 2–9 workdays’ data (median 7 days) and 2–9 non-workdays’ data (median 4 days)

^c^ Calculated as the CNBQ-based value minus the EMA food diary-based value (at the individual level). Paired comparison was made using the paired t-test: * *P* < 0.05, ** *P* < 0.01, *** *P* < 0.001, and **** *P* < 0.0001

^d^ Calculated as mean difference plus-minus 1.96 SD of the difference

^e^ All values were significant (*P* < 0.0001)

^f^ Calculated using the start time of first eating occasion and the start time of last eating occasion

### Bland–Altman plots

**Fig. 3:**
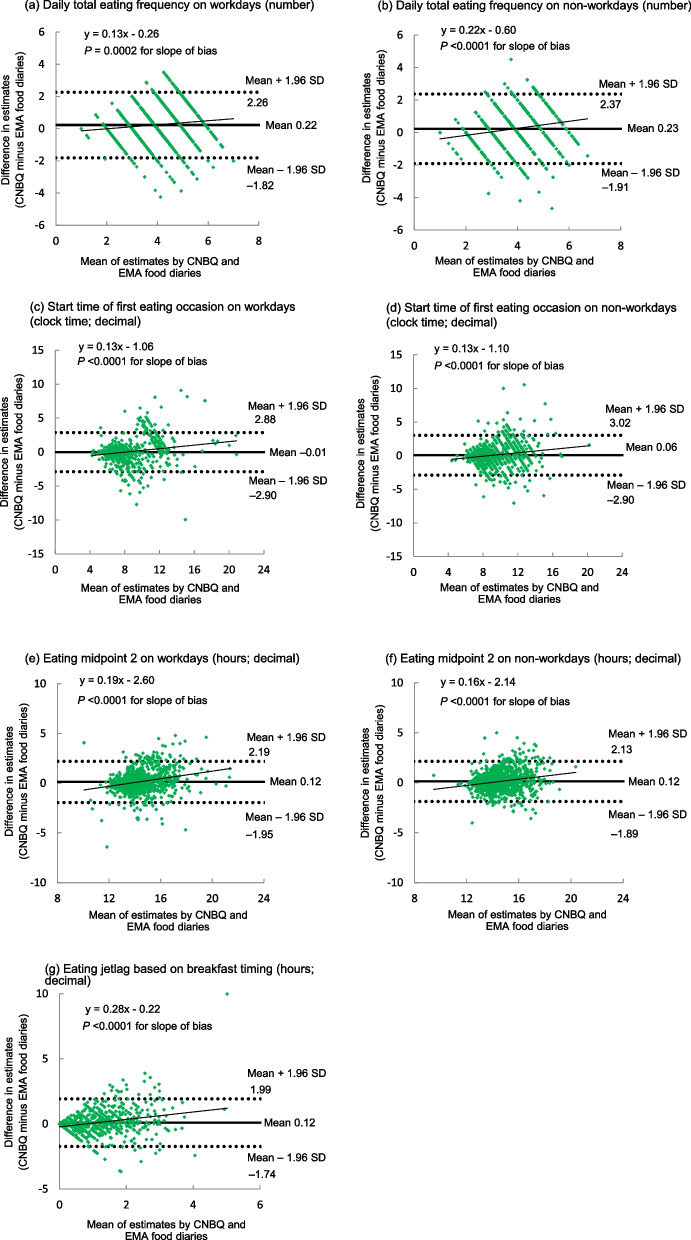
Bland–Altman plots assessing agreement between the CNBQ and EMA food diaries for selected variables. CNBQ, Chrono-Nutrition Behavior Questionnaire; EMA, ecological momentary assessment; SD, standard deviation. All time-related variables are shown in decimal format, with the unit of *clock time; decimal* (e.g., 8.50 means 8:30 AM, while 16.75 means 4:45 PM) or *hours; decimal* (e.g., 2.19 means 2 h 11 min). Eating midpoint 2 was calculated using the start time of first eating occasion and the finish time of last eating occasion. *n* = 1050 except for eating jetlag based on breakfast timing (*n* = 753). The number of days of EMA food diary data was 2–9 (median 7) days for (**a**), (**c**), and (**e**); 2–9 (median 4) days for (**b**), (**d**), and (**f**); and 11 days for (**g**).

Figure 3 shows Bland–Altman plots assessing the agreement between selected variables derived from the CNBQ and EMA food diaries. The magnitude of mean differences was relatively small (i.e., < 9%) for all variables. However, the limits of agreement were generally wide, indicating poor agreement at the individual level (see Tables 2–4 for other variables). There was also an indication of proportional bias between the CNBQ and EMA food diary-based estimates for all the variables examined, in which CNBQ-based estimates tended to be overestimated as the average estimates increased.

## Discussion

To our knowledge, this is the first study to examine the relative validity of a novel questionnaire (CNBQ) designed to collect comprehensive data on chrononutrition-related parameters. Using the 11-day event-based EMA food diaries as a reference method, this study showed that the CNBQ has acceptable relative validity for estimating mean values (i.e., < 10% difference [49]) for the majority of chrononutrition-related parameters examined: 27 (of 33; 82%) workday variables, 25 (of 33; 76%) non-workday variables, and 5 (of 6; 83%) variables based on differences between workdays and non-workdays. These results are generally consistent with several previous studies. For example, Hartman et al. evaluated the relative validity of a 24-h grid approach to assessing eating timing and frequency, in comparison to data derived from up to 6 unannounced, telephone, interviewer-administered 24-h dietary recalls in 626 US adults (64% females) [37]. Mean differences between the 2 methods were < 10% for 8 out of 10 variables examined for both weekdays and weekend days [37]. Similarly, in another study in 249 US adults (75% females), median values of all the 4 food timing variables assessed via recall-based survey questions were similar (< 8% difference) to those derived from up to 14-day paper-based food records [36]. Furthermore, mean differences between estimates derived from a Chrononutrition Questionnaire and those derived from a diary method (a 7-day food diary and a 14-day sleep diary) were < 10% for 13 of 16 workday variables (81%) and 14 of 16 non-workday variables (88%) in a small group (*n* = 58) of general adult population in Australia [29]. Considering large differences in participant characteristics, the test and reference methods, and the variables examined between studies, these consistent findings may suggest that well designed questionnaires may be useful for assessing a number of chrononutrition-related parameters, at least at the group level.

However, our Bland–Altman analyses showed wide limits of agreement, clearly indicating that the ability to obtain accurate estimates at the individual level was generally limited. This is largely consistent with previous studies in the context of chrononutrition-related parameters [29, 36] but also of common dietary variables (nutrient and food intakes) [30, 39–41, 49]. Furthermore, generally similar degrees of both underestimation and overestimation by the CNBQ were apparent for almost all variables, probably resulting in relatively accurate estimates at the group level. This is interesting given that in dietary surveys underreporting, but not overreporting, of energy intake is highly prevalent [56–58]. In fact, it remains unclear whether chrononutrition-related variables (such as timing and frequency of eating) are prone to underpeporting [6], which is possible considering that more frequent eating is positively related to energy intake [46, 59] and later food timing tends to be stigmatized [25]. Further research in this regard is warranted.

The results from previous studies are not consistent in terms of the ability of chrononutrition-related questionnaires to rank individuals in a population. For example, in the US study mentioned above [36], in which 4 food timing variables assessed via recall-based survey questions were examined against those derived from up to 14-day paper-based food records, Kendall’s correlation coefficients were unacceptable (< 0.50 [37]) in all cases, ranging from 0.05 to 0.45. These results may reflect the problem of answering questions such as ‘At what time do you *first start eating* on weekdays/workdays? (includes meals, snacks, and drink meals, but not calorie-free beverages)’ [36]. This type of question remarkably contrasts with those of the majority of previous studies [29, 35, 37] and the present study, in which researchers asked participants to report the timing of each eating occasion and then identified, for example, the first eating occasion after data collection. In contrast, somewhat high correlations (> 0.40; exact values not available) were observed between the timing of breakfast, lunch, and dinner (but not of snacks) derived from the Food Timing Questionnaire and its short version (Food Timing Screener) and a reference method, namely Automated Self-Administered 24-h recall conducted for 7 consecutive days, in a small US study (*n* = 61) [35]. Furthermore, in the US study by Hartman et al. [37], Spearman correlation coefficients between the 24-h grid approach and 24-h recalls were ≥ 0.50 for the majority of weekday variables (6 of 10); however, this was not the case for weekend day variables, as only one variable had a correlation of ≥ 0.50. Higher correlations (≥ 0.50) for workday variables compared with non-workday variables (10 vs 8 out of 16) were also observed in the Australian study by Phoi et al. [29]. These results from Hartman et al. [37] and Phoi et al. [29] may be consistent with the commonly held belief that people generally eat more regularly on weekdays (or workdays) than on weekends (or non-workdays) [37]. Alternatively, these results may also indicate the need to assess a larger number of weekend days or non-workdays to obtain a more representative picture of eating patterns on weekend days or non-workdays (the number of weekend days [37] and non-workdays [29] assessed was not explicitly reported in these studies). Conversely, this study showed that the CNBQ has acceptable relative validity in terms of ranking individuals (i.e., Spearman correlation coefficient ≥ 0.50 [37]) for the majority of chrononutrition-related parameters examined for both workdays (26 variables; 79%) and non-workdays (22 variables; 67%) as well as the variables based on differences between workdays and non-workdays (2 variables; 33%). The relatively large number of non-workdays (mean 4.5 days) assessed in our present study may account for the more encouraging correlation coefficients for many variables, even for non-workdays. This may at least partially help to explain the discrepancies between our present results and those of Hartman et al. [37] and Phoi et al. [29].

Our results that the CNBQ has acceptable relative validity for estimating mean values and ranking individuals for the majority of chrononutrition-related parameters are perhaps not surprising, given that the CNBQ was developed based on careful consideration of the existing tools mentioned above [29, 33–37]. In addition, we speculate that our satisfactory results may be largely owing to the use of pre-specified eating occasion slots which aimed to fit well with the eating patterns of Japanese, namely a relatively stable pattern of frequency, timing, and time spent with regard to meals (breakfast, lunch, and dinner), and infrequent snack consumption [23]. As a result, the potential implications of the present results should only be interpreted in relation to the particular questionnaire (CNBQ) with considering cultural relevance. Specifically, the CNBQ might be applied with little difficulty to populations having an eating pattern similar to Japanese (three main meals, with infrequent snacks particularly at night), e.g., Taiwanese [60]. In contrast, potential usefulness of the CNBQ (without any modification) may be limited in most Western countries where less eating frequency and small and infrequent snacks are not common [13, 22]. Therefore, a prerequisite for adopting and validating the CNBQ in Western populations is to take into account eating patterns (e.g., eating frequency, characteristics of snacking behaviors, and energy intake distribution over 24 h) in the target population, and then make appropriate modifications.

There are several limitations in the present study. First, although conducted in diverse regions (26 of 47 prefectures), the participants consisted of volunteers, not a nationally representative sample of the Japanese population. The participants may have been biased toward greater health consciousness, higher socioeconomic status, or both. For example, the education level and annual household income of the participants were higher than those of a nationally representative sample (education: 54.6% for junior high school or high school, 20.8% for junior college or technical school, and 24.6% for university or higher [61]; annual household income: 45.0% for < 4 million yen, 26.9% for ≥ 4 to < 7 million yen, and 28.0% for ≥ 7 million yen [62]). Meanwhile, the prevalence of current smokers and mean BMI in the present participants were similar to those of the nationally representative sample (males: 27.1% and 23.9 kg/m^2^ (SD 3.6), respectively; females: 7.6% and 22.5 kg/m^2^ (SD 3.7), respectively) [63]. Ideally, further validation should be examined using a more nationally representative sample.

Second, event-based EMA food diaries – the reference method in this study – are ultimately dependent on the self-reporting of participants and thus susceptible to measurement errors due to erroneous recording and potential changes in eating behavior [30, 64, 65]. Nevertheless, while we welcome the recent development of a number of automatic, wearable-based approaches to objectively capture eating events, these are still impractical for use in free-living settings [66]. In fact, the validity of these approaches is quite often examined in comparison with self-reports of eating behaviors [66]. Considered broadly, EMA food diaries appear to be an optimal reference tool which enable the collection of a wide range of information based on actual eating behaviors without relying on memory.

Third, because the data were collected over a narrow time frame (between February and April 2023; late winter and early spring in Japan), and also due to the 1-month time reference period used in the CNBQ, potential seasonal differences in chrononutrition-related parameters, and thus their effect on the validity of the CNBQ, could not be considered in the present study. However, a recent study showed that the minimum number of days required to obtain reliable estimates of meal timing variables (overnight fasting duration, the midpoint of overnight fasting time, number of eating episodes per day, daily period of greatest percentage caloric intake, and late last eating episode) over a 1–3-year period was 3 days [24], suggesting small seasonal differences in these variables. Thus, there is no strong reason to consider that seasonal differences in chrononutrition-related parameters are a serious problem, albeit that further investigation is needed.

Finally, due to the lack of questions on dietary intake in the CNBQ, this study did not include some relevant variables in the context of chrononutrition, such as energy intake distribution across the day, midpoint of energy intake, and period of greatest energy intake consumed (i.e., timing of largest meal) [11, 29]. Note, however, that we recently developed and validated the MDHQ [38–41], a dietary assessment questionnaire specifically designed to assess dietary intake at each eating occasion (breakfast, morning snack, lunch, afternoon snack, dinner, and evening snack), which was incorporated into this study. Thus, combining data from the CNBQ and MDHQ [38–41] allows for the creation of the chrononutrition-related variables described above; examination of relative validity of this innovative approach was beyond the scope of this study and thus would be the next step.

Despite these limitations, the strengths of the present study include the use of EMA food diaries with a large number of recording days (11 days) as the reference method and the comprehensive nature of the CNBQ covering a wide range of chrononutrition-related parameters with clear definitions. This methodological study is novel and highly relevant to the growing interest in temporal patterns of eating and chrononutrition in the discussion of nutrition, health, and chronic disease prevention [9–12]. Specifically, this study suggests that the CNBQ may be useful to investigate the association between chrononutrition-related behaviors and health outcomes in large-scale observational studies as well as intervention trials (e.g., to assess compliance of time-restricted eating). However, the study of chrononutrition cannot be isolated from the reality of dietary intake and quality. It should be fully realized that a better understanding of chrono-nutritional aspects of eating behaviors in relation to health will be obtained when information on dietary intake and quality is available. Thus, the concurrent assessment of chrononutrition behaviors (e.g., by CNBQ or other relevant tools) and dietary intake and quality (eg, by MDHQ [38–41] and other dietary assessment tools) should always be encouraged so that the emerging research field of chrononutrition moves forward to higher levels.

## Conclusions

Compared with 11-day event-based EMA food diaries, the CNBQ, a novel questionnaire designed to collect comprehensive data on chrononutrition-related parameters, showed sufficient relative validity in terms of estimating mean values and ranking individuals for the majority of chrononutrition-related parameters. In contrast, its ability to produce accurate estimates at the individual level was generally limited. Although both the strengths and weaknesses of the CNBQ described in this study should be carefully considered in any setting, the CNBQ may be a promising tool in large chrono-nutritional observational studies and intervention trials where more detailed assessment may not be feasible.

## Supplementary Information

Supplementary Material 1

## Data Availability

The datasets generated and analyzed during the current study are not publicly available but may be made available by the corresponding author on reasonable request and upon approval by the Ethics Committee of the University of Tokyo Faculty of Medicine.

## Abbreviations

BMI: Body mass index
CI: Confidence interval
CNBQ: Chrono-Nutrition Behavior Questionnaire
EMA: Ecological momentary assessment
MDHQ: Meal-based Diet History Questionnaire
SD: Standard deviation
5 W study: Who, What, When, Where, and Why for Healthy Eating study
