# Supplementary material for: Relative validity of the Chrono-Nutrition Behavior Questionnaire (CNBQ) against 11-day event-based ecological momentary assessment diaries of eating
Source: Int J Behav Nutr Phys Act. 2025 Apr 25;22:46. doi: 10.1186/s12966-025-01740-9 (PMC12023641; doi:10.1186/s12966-025-01740-9)
Supplement: Supplementary file 1 — Supplementary Material 1 [file 12966_2025_1740_MOESM1_ESM.docx]

**Relative validity of the Chrono-Nutrition Behavior Questionnaire (CNBQ) against 11-day event-based ecological momentary assessment diaries of eating**

Kentaro Murakami^1*^, Nana Shinozaki^1^, Tracy A. McCaffrey^2^, M. Barbara E. Livingstone^3^ Shizuko Masayasu^4^, and Satoshi Sasaki^1^

^1^ Department of Social and Preventive Epidemiology, School of Public Health, University of Tokyo, Tokyo, Japan

^2^ Department of Nutrition, Dietetics and Food, Monash University, Clayton, Melbourne, Australia

^3^ Nutrition Innovation Centre for Food and Health (NICHE), School of Biomedical Sciences, Ulster University, Coleraine, UK

^4^ Ikurien-Naka, Ibaraki, Japan

* Corresponding author: Kentaro Murakami, Department of Social and Preventive Epidemiology, School of Public Health, University of Tokyo, Tokyo 113-0033, Japan. Tel: +81 3 5841 7872; Fax: +81 3 5841 7873; E-mail: kenmrkm@m.u-tokyo.ac.jp

Supplemental Table 1. Question items in the Chrono-Nutrition Behavior Questionnaire (CNBQ): original Japanese version

| Question ID | Question | Response option |
| --- | --- | --- |
| [Part 1] | --- | --- |
| Instruction for G01 and G02 | このセクションでは、仕事や学校のある日とない日の睡眠・起床習慣に関しておたずねします。  この1か月間のあなたの平均的な「通常」の睡眠習慣をお答えください。 | --- |
| G01 | 過去3か月間に、交代勤務や夜勤をしていましたか？ | いいえ  はい |
| G02 | この1ヶ月間、週に何日、仕事や学校がありましたか？  仕事や学校がない人（専業主婦・主夫の方など）：配偶者が働いている日を「仕事がある日」としてお答えください。配偶者が働いていない、もしくはいない場合は、平日（月～金）を仕事がある日と考えてください。 | 週0日  週1日  週2日  週3日  週4日  週5日  週6日  週7日 |
| [Part 2] | --- | --- |
| Instruction for W01 to W03 | 仕事や学校がある日についておたずねします。  仕事や学校がない人（専業主婦・主夫の方など）：配偶者が働いている日を「仕事がある日」としてお答えください。配偶者が働いていない、もしくはいない場合は、平日（月～金）を仕事がある日と考えてください。 | --- |
| W01 | この1か月、仕事や学校がある日に、あなたは通常、何時に眠りにつきましたか？  ベッドに入った時刻ではなく、眠りについた時刻を、24時間制で回答 （例．24:30→0:30） | 数値（時刻） |
| W02 | この1か月、仕事や学校がある日に、あなたは通常、何時に目を覚ましましたか？  ベッドから出た時刻ではなく、目を覚ました時刻を、24時間制で回答 | 数値（時刻） |
| W03 | この1か月、仕事や学校がある日に、目覚まし時計を使っていましたか？ | いいえ  はい |
| Instruction for W04 to W09 | この1か月間で、仕事や学校がある日の、食事の時刻についておたずねします。 仕事や学校がない人（専業主婦・主夫の方など）：配偶者が働いている日を「仕事がある日」としてお答えください。配偶者が働いていない、もしくはいない場合は、平日（月～金）を仕事がある日と考えてください。 | --- |
| W04 | この1か月間で、仕事や学校がある日には、通常何時ごろに朝食をとっていましたか？  24時間制の半角数字4桁で入力（例：午前7:13→0713）。水や飲み物しかとらないか、食べない場合→0を入力 | 4桁の数字（hhmm） 食べない＝0 |
| W05 | この1か月間で、仕事や学校がある日には、通常何時ごろに午前の間食をとっていましたか？  24時間制の半角数字4桁で入力（例：午前10:25→1025）。水や飲み物しかとらないか、食べない場合 →0を入力。午前の間食を毎日2回以上とる場合→それらを平均した時刻を入力（例：午前10:00と午前11:00に食べる場合、1030） | 4桁の数字（hhmm） 食べない＝0 |
| W06 | この1か月間で、仕事や学校がある日には、通常何時ごろに昼食をとっていましたか？  24時間制の半角数字4桁で入力（例：午後0:38→1238）。水や飲み物しかとらないか、食べない場合→0を入力 | 4桁の数字（hhmm） 食べない＝0 |
| W07 | この1か月間で、仕事や学校がある日には、通常何時ごろに午後の間食をとっていましたか？  24時間制の半角数字4桁で入力（例：午後3:10→1510）。水や飲み物しかとらないか、食べない場合→0を入力。午後の間食を毎日2回以上とる場合→それらを平均した時刻を入力（例：午後3:00と午後5:00に食べる場合、1600） | 4桁の数字（hhmm） 食べない＝0 |
| W08 | この1か月間で、仕事や学校がある日には、通常何時ごろに夕食をとっていましたか？  24時間制の半角数字4桁で入力（例：午後7:15→1915）。水や飲み物しかとらないか、食べない場合→0を入力 | 4桁の数字（hhmm） 食べない＝0 |
| W09 | この1か月間で、仕事や学校がある日には、通常何時ごろに夜間の間食をとっていましたか？  24時間制の半角数字4桁で入力（例：午前0:32→0032）。水や飲み物しかとらないか、食べない場合→0を入力。夜間の間食を毎日2回以上とる場合→それらを平均した時刻を入力（例：午後8:00と午後11:00に食べる場合、2130） | 4桁の数字（hhmm） 食べない＝0 |
| Instruction for W10 to W15 | この1か月間で、仕事や学校がある日の、食事にかかった時間についておたずねします。 仕事や学校がない人（専業主婦・主夫の方など）：配偶者が働いている日を「仕事がある日」としてお答えください。配偶者が働いていない、もしくはいない場合は、平日（月～金）を仕事がある日と考えてください。 | --- |
| W10 | この1か月間で、仕事や学校がある日に、朝食を食べ始めてから食べ終えるまでに通常何分程度かかっていましたか？  半角数字で入力（例：15分→15）。水や飲み物しかとらないか、食べない場合→0を入力 | 数値（分）  食べない＝0 |
| W11 | この1か月間で、仕事や学校がある日に、午前の間食を食べ始めてから食べ終えるまでに通常何分程度かかっていましたか？  半角数字で入力（例：15分→15）。水や飲み物しかとらないか、食べない場合→0を入力。午前の間食を2回以上とる場合→合計の時間を入力 | 数値（分）  食べない＝0 |
| W12 | この1か月間で、仕事や学校がある日に、昼食を食べ始めてから食べ終えるまでに通常何分程度かかっていましたか？  半角数字で入力（例：15分→15）。水や飲み物しかとらないか、食べない場合→0を入力 | 数値（分）  食べない＝0 |
| W13 | この1か月間で、仕事や学校がある日に、午後の間食を食べ始めてから食べ終えるまでに通常何分程度かかっていましたか？  半角数字で入力（例：15分→15）。水や飲み物しかとらないか、食べない場合→0を入力。午後の間食を2回以上とる場合→合計の時間を入力 | 数値（分）  食べない＝0 |
| W14 | この1か月間で、仕事や学校がある日に、夕食を食べ始めてから食べ終えるまでに通常何分程度かかっていましたか？  半角数字で入力（例：15分→15）。水や飲み物しかとらないか、食べない場合→0を入力 | 数値（分）  食べない＝0 |
| W15 | この1か月間で、仕事や学校がある日に、夜間の間食を食べ始めてから食べ終えるまでに通常何分程度かかっていましたか？  半角数字で入力（例：15分→15）。水や飲み物しかとらないか、食べない場合→0を入力。夜間の間食を2回以上とる場合→合計の時間を入力 | 数値（分）  食べない＝0 |
| [Part 3] | --- | --- |
| Instruction for F01 to F03 | 仕事や学校がない日についておたずねします。 仕事や学校がない人（専業主婦・主夫の方など）：配偶者が働いていない日を「仕事がない日」としてお答えください。配偶者が働いていない、もしくはいない場合は、土日を仕事がない日と考えてください。 | --- |
| F01 | この1か月、仕事や学校がない日に、あなたは通常、何時に眠りにつきましたか？  ベッドに入った時刻ではなく、眠りについた時刻を、24時間制で回答 （例．24:30→0:30） | 数値（時刻） |
| F02 | この1か月、仕事や学校がない日に、あなたは通常、何時に目を覚ましましたか？  ベッドから出た時刻ではなく、目を覚ました時刻を、24時間制で回答 | 数値（時刻） |
| F03 | この1か月、仕事や学校がない日に、目覚まし時計を使っていましたか？ | いいえ  はい |
| Instruction for F04 to F09 | この1か月間で、仕事や学校がない日の、食事の時刻についておたずねします。 仕事や学校がない人（専業主婦・主夫の方など）：配偶者が働いていない日を「仕事がない日」としてお答えください。配偶者が働いていない、もしくはいない場合は、土日を仕事がない日と考えてください。 | --- |
| F04 | この1か月間で、仕事や学校がない日には、通常何時ごろに朝食をとっていましたか？  24時間制の半角数字4桁で入力（例：午前7:13→0713）。水や飲み物しかとらないか、食べない場合→0を入力 | 4桁の数字（hhmm） 食べない＝0 |
| F05 | この1か月間で、仕事や学校がない日には、通常何時ごろに午前の間食をとっていましたか？  24時間制の半角数字4桁で入力（例：午前10:25→1025）。水や飲み物しかとらないか、食べない場合→0を入力。午前の間食を毎日2回以上とる場合→それらを平均した時刻を入力（例：午前10:00と午前11:00に食べる場合、1030） | 4桁の数字（hhmm） 食べない＝0 |
| F06 | この1か月間で、仕事や学校がない日には、通常何時ごろに昼食をとっていましたか？  24時間制の半角数字4桁で入力（例：午後0:38→1238）。水や飲み物しかとらないか、食べない場合→0を入力 | 4桁の数字（hhmm） 食べない＝0 |
| F07 | この1か月間で、仕事や学校がない日には、通常何時ごろに午後の間食をとっていましたか？  24時間制の半角数字4桁で入力（例：午後3:10→1510）。水や飲み物しかとらないか、食べない場合→0を入力。午後の間食を毎日2回以上とる場合→それらを平均した時刻を入力（例：午後3:00と午後5:00に食べる場合、1600） | 4桁の数字（hhmm） 食べない＝0 |
| F08 | この1か月間で、仕事や学校がない日には、通常何時ごろに夕食をとっていましたか？  24時間制の半角数字4桁で入力（例：午後7:15→1915）。水や飲み物しかとらないか、食べない場合→0を入力 | 4桁の数字（hhmm） 食べない＝0 |
| F09 | この1か月間で、仕事や学校がない日には、通常何時ごろに夜間の間食をとっていましたか？  24時間制の半角数字4桁で入力（例：午前0:32→0032）。水や飲み物しかとらないか、食べない場合→0を入力。夜間の間食を毎日2回以上とる場合→それらを平均した時刻を入力（例：午後8:00と午後11:00に食べる場合、2130） | 4桁の数字（hhmm） 食べない＝0 |
| Instruction for F10 to F15 | この1か月間で、仕事や学校がない日の、食事にかかった時間についておたずねします。 仕事や学校がない人（専業主婦・主夫の方など）：配偶者が働いていない日を「仕事がない日」としてお答えください。配偶者が働いていない、もしくはいない場合は、土日を仕事がない日と考えてください。 | --- |
| F10 | この1か月間で、仕事や学校がない日に、朝食を食べ始めてから食べ終えるまでに通常何分程度かかっていましたか？  半角数字で入力（例：15分→15）。水や飲み物しかとらないか、食べない場合→0を入力 | 数値（分）  食べない＝0 |
| F11 | この1か月間で、仕事や学校がない日に、午前の間食を食べ始めてから食べ終えるまでに通常何分程度かかっていましたか？  半角数字で入力（例：15分→15）。水や飲み物しかとらないか、食べない場合→0を入力。午前の間食を2回以上とる場合→合計の時間を入力 | 数値（分）  食べない＝0 |
| F12 | この1か月間で、仕事や学校がない日に、昼食を食べ始めてから食べ終えるまでに通常何分程度かかっていましたか？  半角数字で入力（例：15分→15）。水や飲み物しかとらないか、食べない場合→0を入力 | 数値（分）  食べない＝0 |
| F13 | この1か月間で、仕事や学校がない日に、午後の間食を食べ始めてから食べ終えるまでに通常何分程度かかっていましたか？  半角数字で入力（例：15分→15）。水や飲み物しかとらないか、食べない場合→0を入力。午後の間食を2回以上とる場合→合計の時間を入力 | 数値（分）  食べない＝0 |
| F14 | この1か月間で、仕事や学校がない日に、夕食を食べ始めてから食べ終えるまでに通常何分程度かかっていましたか？  半角数字で入力（例：15分→15）。水や飲み物しかとらないか、食べない場合→0を入力 | 数値（分）  食べない＝0 |
| F15 | この1か月間で、仕事や学校がない日に、夜間の間食を食べ始めてから食べ終えるまでに通常何分程度かかっていましたか？  半角数字で入力（例：15分→15）。水や飲み物しかとらないか、食べない場合→0を入力。夜間の間食を2回以上とる場合→合計の時間を入力 | 数値（分）  食べない＝0 |

Participants who answered in question G02 that they worked seven days a week in the previous month were not provided with a series of questions on non-workdays (questions F01–F15).

Supplemental Table 2. Question items in the Chrono-Nutrition Behavior Questionnaire (CNBQ): translated English version

| Question ID | Question | Response option |
| --- | --- | --- |
| [Part 1] | --- | --- |
| Instruction for G01 and G02 | This section asks you about your sleeping and waking habits on days when you are at paid work or school and on days when you are not.  Please consider your average 'normal' sleeping habits over the past month. | --- |
| G01 | Have you worked shifts or night shifts in the past three months? | No  Yes |
| G02 | During the past month, how many days a week did you have paid work or school?  People without a paid job or school (e.g., primary homemakers and caregivers): please consider the days when your partner is engaged in paid work as ‘workdays’ for you. If your partner does not have a paid job or if you do not have a partner, please consider weekdays (Monday to Friday) as ‘workdays’ for you. | 0 day a week  1 day a week  2 days a week  3 days a week  4 days a week  5 days a week  6 days a week  7 days a week |
| [Part 2] | --- | --- |
| Instruction for W01 to W03 | We would like to ask about days when you have paid work or school. People without a paid job or school (e.g., primary homemakers and caregivers): please consider the days when your partner is engaged in paid work as ‘workdays’ for you. If your partner does not have a paid job or if you do not have a partner, please consider weekdays (Monday to Friday) as ‘workdays’ for you. | --- |
| W01 | During the past month, on days when you had paid work or school, what time did you usually fall asleep?  Answer the time when you fell asleep, not when you went to bed, using the 24-hour time format (e.g., 24:30 is 0:30). | Numeric (clock time) |
| W02 | During the past month, on days when you had paid work or school, what time did you usually wake up?  Answer the time you woke up, not the time you got out of bed, using the 24-hour time format. | Numeric (clock time) |
| W03 | In the past month, have you used an alarm clock on days when you had paid work or school? | No  Yes |
| Instruction for W04 to W09 | We would like to ask about your meal timing on days when you have paid work or school during the past month.  People without a paid job or school (e.g., primary homemakers and caregivers): please consider the days when your partner is engaged in paid work as ‘workdays’ for you. If your partner does not have a paid job or if you do not have a partner, please consider weekdays (Monday to Friday) as ‘workdays’ for you. | --- |
| W04 | Over the past month, on days when you had paid work or school, what time did you usually have breakfast?  Enter 4 digits in the 24-hour time format (e.g., 7:13 am is 0713). If you only take water or drinks or do not eat, enter 0. | 4-digit number (hh:mm) I did not eat breakfast = 0 |
| W05 | Over the past month, on days when you had paid work or school, what time did you usually have your morning snack?  Enter 4 digits in the 24-hour time format (e.g., 10:25 am is 1025). If you only take water or drinks or do not eat, enter 0. If you take two or more morning snacks every day, enter the average time of these (e.g., if you eat at 10:00 am and 11:00 am, enter 1030). | 4-digit number (hh:mm) I did not eat morning snack = 0 |
| W06 | Over the past month, on days when you had paid work or school, what time did you usually have lunch?  Enter 4 digits in the 24-hour time format (e.g., 0:38 pm is 1238). If you only take water or drinks or do not eat, enter 0. | 4-digit number (hh:mm) I did not eat lunch = 0 |
| W07 | Over the past month, on days when you had paid work or school, what time did you usually have your afternoon snack?  Enter 4 digits in the 24-hour time format (e.g., 3:10 pm is 1510). If you only take water or drinks or do not eat, enter 0. If you take two or more afternoon snacks every day, enter the average time of these (e.g., if you eat at 3:00 pm and 5:00 pm, enter 1600). | 4-digit number (hh:mm) I did not eat afternoon snack = 0 |
| W08 | Over the past month, on days when you had paid work or school, what time did you usually have dinner?  Enter 4 digits in the 24-hour time format (e.g., 7:15 pm is 1915). If you only take water or drinks or do not eat, enter 0. | 4-digit number (hh:mm) I did not eat dinner = 0 |
| W09 | Over the past month, on days when you had paid work or school, what time did you usually have your evening snack?  Enter 4 digits in the 24-hour time format (e.g., 0:32 am is 0032). If you only take water or drinks or do not eat, enter 0. If you take two or more evening snacks every day, enter the average time of these (e.g., if you eat at 8:00 pm and 11:00 pm, enter 2130). | 4-digit number (hh:mm) I did not eat evening snack = 0 |
| Instruction for W10 to W15 | We would like to ask about the time you spent eating on days when you have paid work or school during the past month.  People without a paid job or school (e.g., primary homemakers and caregivers): please consider the days when your partner is engaged in paid work as ‘workdays’ for you. If your partner does not have a paid job or if you do not have a partner, please consider weekdays (Monday to Friday) as ‘workdays’ for you. | --- |
| W10 | In the past month, on days when you had paid work or school, how long did it usually take you to start and finish breakfast?  Enter numbers (e.g., 15 minutes is 15). If you only take water or drinks or do not eat, enter 0. | Numeric (minutes)  I did not eat breakfast = 0 |
| W11 | In the past month, on days when you had paid work or school, how long did it usually take you to start and finish your morning snack?  Enter numbers (e.g., 15 minutes is 15). If you only take water or drinks or do not eat, enter 0. If you take two or more morning snacks, enter the total time. | Numeric (minutes)  I did not eat morning snack = 0 |
| W12 | In the past month, on days when you had paid work or school, how long did it usually take you to start and finish lunch?  Enter numbers (e.g., 15 minutes is 15). If you only take water or drinks or do not eat, enter 0. | Numeric (minutes)  I did not eat lunch = 0 |
| W13 | In the past month, on days when you had paid work or school, how long did it usually take you to start and finish your afternoon snack?  Enter numbers (e.g., 15 minutes is 15). If you only take water or drinks or do not eat, enter 0. If you take two or more afternoon snacks, enter the total time. | Numeric (minutes)  I did not eat afternoon snack = 0 |
| W14 | In the past month, on days when you had paid work or school, how long did it usually take you to start and finish dinner?  Enter numbers (e.g., 15 minutes is 15). If you only take water or drinks or do not eat, enter 0. | Numeric (minutes)  I did not eat dinner = 0 |
| W15 | In the past month, on days when you had paid work or school, how long did it usually take you to start and finish your evening snack?  Enter numbers (e.g., 15 minutes is 15). If you only take water or drinks or do not eat, enter 0. If you take two or more evening snacks, enter the total time. | Numeric (minutes)  I did not eat evening snack = 0 |
| [Part 3] | --- | --- |
| Instruction for F01 to F03 | We would like to ask about days when you do not have paid work or school. People without a paid job or school (e.g., primary homemakers and caregivers): please consider the days when your partner is not engaged in paid work as ‘work-free days’ for you. If your partner does not have a paid job or if you do not have a partner, please consider Saturdays and Sundays as ‘work-free days’ for you. | --- |
| F01 | During the past month, on days when you did not have paid work or school, what time did you usually fall asleep?  Answer the time when you fell asleep, not when you went to bed, using the 24-hour time format (e.g., 24:30 is 0:30). | Numeric (clock time) |
| F02 | During the past month, on days when you did not have paid work or school, what time did you usually wake up?  Answer the time you woke up, not the time you got out of bed, using the 24-hour time format. | Numeric (clock time) |
| F03 | In the past month, have you used an alarm clock on days when you did not have paid work or school? | No  Yes |
| Instruction for F04 to F09 | We would like to ask about your meal timing on days when you do not have paid work or school during the past month.  People without a paid job or school (e.g., primary homemakers and caregivers): please consider the days when your partner is not engaged in paid work as ‘work-free days’ for you. If your partner does not have a paid job or if you do not have a partner, please consider Saturdays and Sundays as ‘work-free days’ for you. | --- |
| F04 | Over the past month, on days when you did not have paid work or school, what time did you usually have breakfast?  Enter 4 digits in the 24-hour time format (e.g., 7:13 am is 0713). If you only take water or drinks or do not eat, enter 0. | 4-digit number (hh:mm) I did not eat breakfast = 0 |
| F05 | Over the past month, on days when you did not have paid work or school, what time did you usually have your morning snack?  Enter 4 digits in the 24-hour time format (e.g., 10:25 am is 1025). If you only take water or drinks or do not eat, enter 0. If you take two or more morning snacks every day, enter the average time of these (e.g., if you eat at 10:00 am and 11:00 am, enter 1030). | 4-digit number (hh:mm) I did not eat morning snack = 0 |
| F06 | Over the past month, on days when you did not have paid work or school, what time did you usually have lunch?  Enter 4 digits in the 24-hour time format (e.g., 0:38 pm is 1238). If you only take water or drinks or do not eat, enter 0. | 4-digit number (hh:mm) I did not eat lunch = 0 |
| F07 | Over the past month, on days when you did not have paid work or school, what time did you usually have your afternoon snack?  Enter 4 digits in the 24-hour time format (e.g., 3:10 pm is 1510). If you only take water or drinks or do not eat, enter 0. If you take two or more afternoon snacks every day, enter the average time of these (e.g., if you eat at 3:00 pm and 5:00 pm, enter 1600). | 4-digit number (hh:mm) I did not eat afternoon snack = 0 |
| F08 | Over the past month, on days when you did not have paid work or school, what time did you usually have dinner?  Enter 4 digits in the 24-hour time format (e.g., 7:15 pm is 1915). If you only take water or drinks or do not eat, enter 0. | 4-digit number (hh:mm) I did not eat dinner = 0 |
| F09 | Over the past month, on days when you did not have paid work or school, what time did you usually have your evening snack?  Enter 4 digits in the 24-hour time format (e.g., 0:32 am is 0032). If you only take water or drinks or do not eat, enter 0. If you take two or more evening snacks every day, enter the average time of these (e.g., if you eat at 8:00 pm and 11:00 pm, enter 2130). | 4-digit number (hh:mm) I did not eat evening snack = 0 |
| Instruction for F010 to F15 | We would like to ask about the time you spent eating on days when you do not have paid work or school during the past month.  People without a paid job or school (e.g., primary homemakers and caregivers): please consider the days when your partner is not engaged in paid work as ‘work-free days’ for you. If your partner does not have a paid job or if you do not have a partner, please consider Saturdays and Sundays as ‘work-free days’ for you. | --- |
| F10 | In the past month, on days when you did not have paid work or school, how long did it usually take you to start and finish breakfast?  Enter numbers (e.g., 15 minutes is 15). If you only take water or drinks or do not eat, enter 0. | Numeric (minutes)  I did not eat breakfast = 0 |
| F11 | In the past month, on days when you did not have paid work or school, how long did it usually take you to start and finish your morning snack?  Enter numbers (e.g., 15 minutes is 15). If you only take water or drinks or do not eat, enter 0. If you take two or more morning snacks, enter the total time. | Numeric (minutes)  I did not eat morning snack = 0 |
| F12 | In the past month, on days when you did not have paid work or school, how long did it usually take you to start and finish lunch?  Enter numbers (e.g., 15 minutes is 15). If you only take water or drinks or do not eat, enter 0. | Numeric (minutes)  I did not eat lunch = 0 |
| F13 | In the past month, on days when you did not have paid work or school, how long did it usually take you to start and finish your afternoon snack?  Enter numbers (e.g., 15 minutes is 15). If you only take water or drinks or do not eat, enter 0. If you take two or more afternoon snacks, enter the total time. | Numeric (minutes)  I did not eat afternoon snack = 0 |
| F14 | In the past month, on days when you did not have paid work or school, how long did it usually take you to start and finish dinner?  Enter numbers (e.g., 15 minutes is 15). If you only take water or drinks or do not eat, enter 0. | Numeric (minutes)  I did not eat dinner = 0 |
| F15 | In the past month, on days when you did not have paid work or school, how long did it usually take you to start and finish your evening snack?  Enter numbers (e.g., 15 minutes is 15). If you only take water or drinks or do not eat, enter 0. If you take two or more evening snacks, enter the total time. | Numeric (minutes)  I did not eat evening snack = 0 |

Participants who answered in question G02 that they worked seven days a week in the previous month were not provided with a series of questions on non-workdays (questions F01–F15).

Supplemental Table 3. Description of sleep and temporal patterns of eating variables used in this study

| Variable | CNBQ | 11-day EMA food diaries |
| --- | --- | --- |
| 1) Sleep variables |  |  |
| 1-1. Sleep time (clock time) | Local time of falling asleep (i.e., sleep onset; hh:mm). Answered for workdays (question W01) and non-workdays (question F01) separately. | Local time of preparing to sleep (hh:mm) plus the length of time that it takes to accomplish the transition from full wakefulness to sleep (i.e., sleep onset latency; asked in min). Mean values were calculated for workdays and non-workdays separately. |
| 1-2. Wake time (clock time) | Local time of waking up (hh:mm). Answered on workdays (question W02) and non-workdays (question F02) separately. | Local time of waking up (hh:mm). Mean values were calculated for workdays and non-workdays separately. |
| 1-3. Sleep duration (hours/day) | [wake time; variable 1-2] − [sleep time; variable 1-1]. Calculated for workdays and non-workdays separately. | [wake time; variable 1-2] − [sleep time; variable 1-1]. Mean values were calculated for workdays and non-workdays separately. |
| 1-4. Mid-sleep time (clock time) | [sleep time; variable 1-1] + [sleep duration; variable 1-3]/2. Calculated for workdays and non-workdays separately. | [sleep time; variable 1-1] + [sleep duration; variable 1-3]/2. Mean values calculated for workdays and non-workdays separately. |
| 2) Daily eating frequency (number) |  |  |
| 2-1. Meals | Sum of the number of valid answers on the start time of breakfast, lunch, and dinner (possible numbers: 0, 1, 2, and 3). Calculated for workdays (using questions W04, W06, and W08) and non-workdays (using questions F04, F06, and F08) separately. | Sum of the number of mentioning breakfast, lunch, and dinner. Mean values were calculated for workdays and non-workdays separately. |
| 2-2. Snacks | Sum of the number of valid answers on the start time of morning snack, afternoon snack, and night snack (possible numbers: 0, 1, 2, and 3). Calculated for workdays (using questions W05, W07, and W09) and non-workdays (using questions F05, F07, and F09) separately. | Sum of the number of mentioning snacks. Mean values were calculated for workdays and non-workdays separately. |
| 2-3. Total | [daily eating frequency of meals; variable 2-1] + [daily eating frequency of snacks; variable 2-2] (possible numbers: 0, 1, 2, 3, 4, 5, and 6). Calculated for workdays and non-workdays separately. | [daily eating frequency of meals; variable 2-1] + [daily eating frequency of snacks; variable 2-2]. Mean values were calculated for workdays and non-workdays separately. |
| 3) Start time of eating (clock time) |  |  |
| 3-1. First eating occasion | Earliest local time (hh:mm) of the start of first meal or first snack (see variables 3-3 and 3-5). Determined for workdays and non-workdays separately. There were no participants with missing information on this variable. | For each day, local time (hh:mm) of the start of first eating occasion was determined as either the start of first meal or first snack (see variables 3-3 and 3-5). Then, mean values were calculated for workdays and non-workdays separately. There were no participants with missing information on this variable. |
| 3-2. Last eating occasion | Latest local time (hh:mm) of the start of last meal or last snack (see variables 3-4 and 3-6). Determined for workdays and non-workdays separately. There were no participants with missing information on this variable. | For each day, local time (hh:mm) of the start of last eating occasion was determined as either the start of last meal or last snack (see variables 3-4 and 3-6). Then, mean values were calculated for workdays and non-workdays separately. There were no participants with missing information on this variable. |
| 3-3. First meal | Earliest local time (hh:mm) of the start of eating breakfast, lunch, or dinner. Determined for workdays (using questions W04, W06, and W08) and non-workdays (using questions F04, F06, and F08) separately. There were no participants with missing information on this variable. | For each day, earliest local time of meal (hh:mm) was determined on the basis of start time of eating breakfast, lunch, and dinner. Then, mean values were calculated for workdays and non-workdays separately. There were no participants with missing information on this variable. |
| 3-4. Last meal | Latest local time (hh:mm) of the start of eating breakfast, lunch, or dinner. Determined for workdays (using questions W04, W06, and W08) and non-workdays (using questions F04, F06, and F08) separately. There were no participants with missing information on this variable. | For each day, latest local time of meal (hh:mm) was determined on the basis of start time of eating breakfast, lunch, and dinner. Then, mean values were calculated for workdays and non-workdays separately. There were no participants with missing information on this variable. |
| 3-5. First snack | Earliest local time (hh:mm) of the start of eating morning snack, afternoon snack, or night snack. Determined for workdays (using questions W05, W07, and W09) and non-workdays (using questions F05, F07, and F09) separately. There were some participants with missing information on this variable. | For each day, earliest local time of snack (hh:mm) was determined on the basis of start time of snacks. For workdays and non-workdays separately, mean values were calculated using data on snack-consumption days; there were some participants with missing values on this variable. |
| 3-6. Last snack | Latest local time (hh:mm) of the start of eating morning snack, afternoon snack, or night snack. Determined for workdays (using questions W05, W07, and W09) and non-workdays (using questions F05, F07, and F09) separately. There were no participants with missing information on this variable. | For each day, latest local time of snack (hh:mm) was determined on the basis of start time of snacks. For workdays and non-workdays separately, mean values were calculated using data on snack-consumption days; there were some participants with missing values on this variable. |
| 3-7. Breakfast | Local time of start of eating breakfast (hh:mm). Answered for workdays (question W04) and non-workdays (question F04) separately. Missing values were assigned for participants who answered no consumption of breakfast. | Local time of start of eating breakfast (hh:mm). For workdays and non-workdays separately, mean values were calculated using data on breakfast-consumption days; there were some participants with missing values on this variable. |
| 3-8. Lunch | Local time of start of eating lunch (hh:mm). Answered for workdays (question W06) and non-workdays (question F06) separately. Missing values were assigned for participants who answered no consumption of lunch. | Local time of start of eating lunch (hh:mm). For workdays and non-workdays separately, mean values were calculated using data on lunch-consumption days; there were some participants with missing values on this variable. |
| 3-9. Dinner | Local time of start of eating dinner (hh:mm). Answered for workdays (question W08) and non-workdays (question F08) separately. Missing values were assigned for participants who answered no consumption of dinner. | Local time of start of eating dinner (hh:mm). For workdays and non-workdays separately, mean values were calculated using data on dinner-consumption days; there were some participants with missing values on this variable. |
| 4) Duration of eating occasion (minutes) |  |  |
| 4-1. First eating occasion | First eating occasion was determined on the basis of earliest local time (hh:mm) of the start of first meal or first snack (see variable 3-1). Then, time spent on first eating occasion (in min) was derived from the response to first meal or first snack (see variables 4-3 and 4-5). This was done for workdays and non-workdays separately. There were no participants with missing information on this variable. | For each day, first eating occasion was determined on the basis of earliest local time (hh:mm) of the start of first meal or first snack (see variable 3-1). Time spent on first eating occasion was calculated as the local time of finish time (hh:mm) minus start time (hh:mm) of either first meal or first snack (see variables 4-3 and 4-5). Then, mean values were calculated for workdays and non-workdays separately. There were no participants with missing information on this variable. |
| 4-2. Last eating occasion | Last eating occasion was determined on the basis of latest local time (hh:mm) of the start of last meal or last snack (see variable 3-2). Then, time spent on last eating occasion (in min) was derived from the response to last meal or last snack (see variables 4-4 and 4-6). This was done for workdays and non-workdays separately. There were no participants with missing information on this variable. | For each day, last eating occasion was determined on the basis of latest local time (hh:mm) of the start of last meal or last snack (see variable 3-2). Time spent on last eating occasion was calculated as the local time of finish time (hh:mm) minus start time (hh:mm) of either last meal or last snack (see variables 4-4 and 4-6). Then, mean values were calculated for workdays and non-workdays separately. There were no participants with missing information on this variable. |
| 4-3. First meal | First meal was determined on the basis of start time of first meal, which may be breakfast, lunch, or dinner (see variable 3-3). Then, time spent on eating the first meal (in min) was derived from the answer on either breakfast, lunch, or dinner (see variables 4-7, 4-8, and 4-9). This was done for workdays and non-workdays separately. There were no participants with missing information on this variable. | For each day, first meal was determined on the basis of start time of first meal, which may be breakfast, lunch, or dinner (see variable 3-3). Time spent on eating first meal was calculated as the local time of finish time (hh:mm) minus start time (hh:mm) of eating either breakfast, lunch, or dinner (see variables 4-7, 4-8, and 4-9). Then, mean values were calculated for workdays and non-workdays separately. There were no participants with missing information on this variable. |
| 4-4. Last meal | Last meal was determined on the basis of start time of last meal, which may be breakfast, lunch, or dinner (see variable 3-4). Then, time spent on eating the last meal (in min) was derived from the answer on either breakfast, lunch, or dinner (see variables 4-7, 4-8, and 4-9). This was done for workdays and non-workdays separately. There were no participants with missing information on this variable. | For each day, last meal was determined on the basis of start time of last meal, which may be breakfast, lunch, or dinner (see variable 3-4). Time spent on eating last meal was calculated as the local time of finish time (hh:mm) minus start time (hh:mm) of eating either breakfast, lunch, or dinner (see variables 4-7, 4-8, and 4-9). Then, mean values were calculated for workdays and non-workdays separately. There were no participants with missing information on this variable. |
| 4-5. First snack | First snack was determined on the basis of start time of first snack, which may be morning snack, afternoon snack, or night snack (see variable 3-5). Then, time spent on eating the first snack (in min) was derived from the answer on either morning snack, afternoon snack, or night snack (questions W11, W13, W15, F11, F13, and F15). This was done for workdays and non-workdays separately. There were some participants with missing information on this variable. | For each day, first snack was determined on the basis of earliest start time (see variable 3-5). Time spent on eating first snack was calculated as the local time of finish time (hh:mm) minus start time (hh:mm) of eating the snack event. For workdays and non-workdays separately, mean values were calculated using data on snack-consumption days; there were some participants with missing values on this variable. |
| 4-6. Last snack | Last snack was determined on the basis of start time of last snack, which may be morning snack, afternoon snack, or night snack (see variable 3-6). Then, time spent on eating the last snack (in min) was derived from the answer on either morning snack, afternoon snack, or night snack (questions W11, W13, W15, F11, F13, and F15). This was done for workdays and non-workdays separately. There were some participants with missing information on this variable. | For each day, last snack was determined on the basis of latest start time (see variable 3-6). Time spent on eating last snack was calculated as the local time of finish time (hh:mm) minus start time (hh:mm) of eating the snack event. For workdays and non-workdays separately, mean values were calculated using data on snack-consumption days; there were some participants with missing values on this variable. |
| 4-7. Breakfast | Time spent on eating breakfast (in min). Answered for workdays (question W10) and non-workdays (question F10) separately. Missing values were assigned for participants who answered no consumption of breakfast (to these questions). | For each day in which consumption of breakfast was mentioned, time spent on eating breakfast was calculated as the local time of finish time (hh:mm) minus start time (hh:mm) of eating. For workdays and non-workdays separately, mean values were calculated using data on breakfast-consumption days; there were some participants with missing values on this variable. |
| 4-8. Lunch | Time spent on eating lunch (in min). Answered for workdays (question W12) and non-workdays (question F12) separately. Missing values were assigned for participants who answered no consumption of lunch (to these questions). | For each day in which consumption of lunch was mentioned, time spent on eating lunch was calculated as the local time of finish time (hh:mm) minus start time (hh:mm) of eating. For workdays and non-workdays separately, mean values were calculated using data on lunch-consumption days; there were some participants with missing values on this variable. |
| 4-9. Dinner | Time spent on eating dinner (in min). Answered for workdays (question W14) and non-workdays (question F14) separately. Missing values were assigned for participants who answered no consumption of dinner (to these questions). | For each day in which consumption of dinner was mentioned, time spent on eating dinner was calculated as the local time of finish time (hh:mm) minus start time (hh:mm) of eating. For workdays and non-workdays separately, mean values were calculated using data on dinner-consumption days; there were some participants with missing values on this variable. |
| 5) Eating window |  |  |
| 5-1. Duration of eating window 1 (hours) | [start time of last eating occasion; variable 3-2] – [start time of first eating occasion; variable 3-1]. Calculated for workdays and non-workdays separately. | [start time of last eating occasion; variable 3-2] – [start time of first eating occasion; variable 3-1]. Mean values were calculated for workdays and non-workdays separately. |
| 5-2. Duration of eating window 2 (hours) | [start time of last eating occasion; variable 3-2] + [time spent on last eating occasion; variable 4-2] – [start time of first eating occasion; variable 3-1]. Calculated for workdays and non-workdays separately. | [start time of last eating occasion; variable 3-2] + [time spent on last eating occasion; variable 4-2] – [start time of first eating occasion; variable 3-1]. Mean values were calculated for workdays and non-workdays separately. |
| 5-3. Eating midpoint 1 (hours) | [start time of first eating occasion; variable 3-1] + [duration of eating window 1; variable 5-1]/2. Calculated for workdays and non-workdays separately. | [start time of first eating occasion; variable 3-1] + [duration of eating window 1; variable 5-1]/2. Mean values were calculated for workdays and non-workdays separately. |
| 5-4. Eating midpoint 2 (hours) | [start time of first eating occasion; variable 3-1] + [duration of eating window 2; variable 5-2]/2. Calculated for workdays and non-workdays separately. | [start time of first eating occasion; variable 3-1] + [duration of eating window 2; variable 5-2]/2. Mean values were calculated for workdays and non-workdays separately. |
| 6) Time interval between sleep and eating (hours) |  |  |
| 6-1. Wake time and first eating occasion | [start time of first eating occasion; variable 3-1] – [wake time; variable 1-2]. Calculated for workdays and non-workdays separately. | [start time of first eating occasion; variable 3-1] – [wake time; variable 1-2]. Mean values were calculated for workdays and non-workdays separately. |
| 6-2. Wake time and first meal | [start time of first meal; variable 3-3] – [wake time; variable 1-2]. Calculated for workdays and non-workdays separately. | [start time of first meal; variable 3-3] – [wake time; variable 1-2]. Mean values were calculated for workdays and non-workdays separately. |
| 6-3. Last eating occasion and sleep time | [sleep time; variable 1-1] – [start time of last eating occasion; variable 4-2]. Calculated for workdays and non-workdays separately. | [sleep time; variable 1-1] – [start time of last eating occasion; variable 4-2]. Mean values were calculated for workdays and non-workdays separately. |
| 6-4. Last meal and sleep time | [sleep time; variable 1-1] – [start time of last meal; variable 4-4]. Calculated for workdays and non-workdays separately. | [sleep time; variable 1-1] – [start time of last meal; variable 4-4]. Mean values were calculated for workdays and non-workdays separately. |

CNBQ, Chrono-Nutrition Behavior Questionnaire; EMA, ecological momentary assessment.

For EMA food diaries, all eating occasions consisting of beverages or water only were excluded. Question items included in the CNBQ are shown in Supplemental Tables 1 (in Japanese) and 2 (in English).

Supplemental Table 4. Sleep and temporal patterns of eating variables on workdays in male participants ^a^

|  | CNBQ | | EMA food diaries ^b^ | | Paired | Mean | Limit of | Spearman |
| --- | --- | --- | --- | --- | --- | --- | --- | --- |
|  | n | Mean (SD) | n | Mean (SD) | n | difference ^c^ | Agreement ^d^ | correlation ^e^ |
| Sleep variables |  |  |  |  |  |  |  |  |
| Sleep time (clock time; decimal) | 532 | 23.51 (1.75) | 532 | 23.50 (1.47) | 532 | 0.01 | -3.33, 3.35 | 0.71 |
| Wake time (clock time; decimal) | 532 | 6.29 (1.24) | 532 | 6.47 (1.32) | 532 | -0.18**** | -2.02, 1.66 | 0.75 |
| Daily sleep duration (hours; decimal) | 532 | 6.69 (1.22) | 532 | 6.61 (1.08) | 532 | 0.08 | -1.80, 1.96 | 0.65 |
| Mid-sleep time (clock time; decimal) | 532 | 3.08 (1.92) | 532 | 3.34 (1.65) | 532 | -0.26**** | -3.03, 2.51 | 0.76 |
| Daily eating frequency (number) |  |  |  |  |  |  |  |  |
| Meals | 532 | 2.75 (0.47) | 532 | 2.73 (0.43) | 532 | 0.02 | -0.54, 0.58 | 0.74 |
| Snacks | 532 | 1.02 (0.98) | 532 | 0.82 (0.84) | 532 | 0.20**** | -1.70, 2.10 | 0.48 |
| Total | 532 | 3.77 (1.13) | 532 | 3.55 (0.97) | 532 | 0.22**** | -1.82, 2.26 | 0.53 |
| Start time of eating (clock time; decimal) |  |  |  |  |  |  |  |  |
| First eating occasion | 532 | 8.24 (2.65) | 532 | 8.30 (2.44) | 532 | -0.05 | -2.97, 2.87 | 0.81 |
| Last eating occasion | 532 | 20.42 (1.81) | 532 | 20.24 (1.31) | 532 | 0.18** | -2.74, 3.10 | 0.63 |
| First meal | 532 | 8.31 (2.78) | 532 | 8.46 (2.58) | 532 | -0.15* | -2.96, 2.66 | 0.81 |
| Last meal | 532 | 19.58 (1.43) | 532 | 19.73 (1.22) | 532 | -0.15** | -2.20, 1.90 | 0.72 |
| First snack | 333 | 16.29 (4.68) | 428 | 16.41 (3.95) | 304 | -0.08 | -9.51, 9.35 | 0.40 |
| Last snack | 333 | 19.85 (3.24) | 428 | 18.51 (3.25) | 304 | 1.29**** | -5.64, 8.22 | 0.35 |
| Breakfast | 417 | 7.02 (0.98) | 473 | 7.25 (1.07) | 414 | -0.12*** | -1.46, 1.22 | 0.74 |
| Lunch | 518 | 12.45 (0.89) | 527 | 12.57 (0.77) | 517 | -0.10** | -1.59, 1.39 | 0.63 |
| Dinner | 528 | 19.64 (1.27) | 532 | 19.81 (1.16) | 528 | -0.17**** | -1.84, 1.50 | 0.74 |
| Duration of eating occasion (minutes; decimal) |  |  |  |  |  |  |  |  |
| First eating occasion | 532 | 13.45 (6.69) | 532 | 10.61 (4.46) | 532 | 2.83**** | -9.97, 15.63 | 0.40 |
| Last eating occasion | 532 | 19.35 (20.26) | 532 | 19.65 (10.35) | 532 | -0.31 | -41.53, 40.91 | 0.26 |
| First meal | 532 | 13.45 (6.69) | 532 | 13.61 (7.14) | 532 | -0.16 | -13.87, 13.55 | 0.56 |
| Last meal | 532 | 27.05 (18.03) | 532 | 31.05 (19.42) | 532 | -4.01**** | -37.02, 29.00 | 0.60 |
| First snack | 324 | 10.85 (12.21) | 428 | 13.86 (22.50) | 298 | -2.30* | -37.04, 32.44 | 0.49 |
| Last snack | 324 | 13.27 (16.64) | 428 | 15.41 (24.06) | 298 | -1.74 | -41.73, 38.25 | 0.45 |
| Breakfast | 420 | 12.90 (6.64) | 473 | 11.99 (5.73) | 417 | 0.55 | -10.81, 11.91 | 0.60 |
| Lunch | 520 | 15.85 (5.91) | 527 | 16.56 (6.93) | 519 | -0.53* | -11.65, 10.59 | 0.59 |
| Dinner | 527 | 27.18 (18.06) | 532 | 31.03 (19.42) | 527 | -3.85**** | -36.79, 29.09 | 0.60 |
| Eating window |  |  |  |  |  |  |  |  |
| Duration of eating window 1 (hours; decimal)^f^ | 532 | 12.18 (3.00) | 532 | 11.94 (2.58) | 532 | 0.23* | -3.87, 4.33 | 0.71 |
| Duration of eating window 2 (hours; decimal)^g^ | 532 | 12.50 (2.97) | 532 | 12.27 (2.59) | 532 | 0.23* | -3.94, 4.40 | 0.70 |
| Eating midpoint 1 (clock time; decimal)^f^ | 532 | 14.33 (1.70) | 532 | 14.27 (1.47) | 532 | 0.06 | -2.02, 2.14 | 0.78 |
| Eating midpoint 2 (clock time; decimal)^g^ | 532 | 14.49 (1.72) | 532 | 14.43 (1.49) | 532 | 0.06 | -2.04, 2.16 | 0.78 |
| Time interval between sleep and eating (hours;  decimal) |  |  |  |  |  |  |  |  |
| Wake time and first eating occasion | 532 | 1.98 (2.58) | 532 | 1.93 (2.32) | 532 | 0.04 | -2.79, 2.87 | 0.75 |
| Wake time and first meal | 532 | 2.05 (2.71) | 532 | 2.06 (2.44) | 532 | -0.01 | -2.77, 2.75 | 0.75 |
| Last eating occasion and sleep time | 532 | 2.88 (1.68) | 532 | 3.14 (1.22) | 532 | -0.26*** | -3.30, 2.78 | 0.50 |
| Last meal and sleep time | 532 | 3.56 (1.64) | 532 | 3.44 (1.35) | 532 | 0.12* | -2.38, 2.62 | 0.69 |

CNBQ, Chrono-Nutrition Behavior Questionnaire; EMA, ecological momentary assessment; SD, standard deviation.

^a^ Meals included breakfast, lunch, and dinner.

^b^ Based on 2–9 days’ data (median 7 days). All time-related variables are shown in decimal format, with the unit of *clock time; decimal* (e.g., 23.51 means 11:31 PM, while 6.29 means 6:17 AM); *hours; decimal* (e.g., 6.69 means a duration of 6 h 41 min); or *minutes; decimal* (e.g., 13.45 means a duration of 13 min 27 sec).

^c^ Calculated as the CNBQ-based value minus the EMA food diary-based value (at the individual level). Paired comparison was made using the paired t-test: * P <0.05, ** P <0.01, *** P <0.001, and **** P <0.0001.

^d^ Calculated as mean difference plus-minus 1.96 SD of the difference.

^e^ All values were significant (P <0.0001).

^f^ Calculated using the start time of first eating occasion and the start time of last eating occasion.

^g^ Calculated using the start time of first eating occasion and the finish time of last eating occasion.

Supplemental Table 5. Sleep and temporal patterns of eating variables on workdays in female participants ^a^

|  | CNBQ | | EMA food diaries ^b^ | | Paired | Mean | Limit of | Spearman |
| --- | --- | --- | --- | --- | --- | --- | --- | --- |
|  | n | Mean (SD) | n | Mean (SD) | n | difference ^c^ | Agreement ^d^ | correlation ^e^ |
| Sleep variables |  |  |  |  |  |  |  |  |
| Sleep time (clock time; decimal) | 518 | 23.51 (1.39) | 518 | 23.62 (1.27) | 518 | -0.11 | -2.96, 2.74 | 0.72 |
| Wake time (clock time; decimal) | 518 | 6.18 (1.10) | 518 | 6.28 (1.09) | 518 | -0.10** | -1.44, 1.24 | 0.82 |
| Daily sleep duration (hours; decimal) | 518 | 6.62 (1.10) | 518 | 6.45 (0.97) | 518 | 0.17**** | -1.61, 1.95 | 0.61 |
| Mid-sleep time (clock time; decimal) | 518 | 2.96 (1.61) | 518 | 3.13 (1.20) | 518 | -0.17** | -2.61, 2.27 | 0.81 |
| Daily eating frequency (number) |  |  |  |  |  |  |  |  |
| Meals | 518 | 2.82 (0.43) | 518 | 2.82 (0.34) | 518 | 0.00 | -0.58, 0.58 | 0.63 |
| Snacks | 518 | 1.40 (0.95) | 518 | 1.17 (0.92) | 518 | 0.23**** | -1.73, 2.19 | 0.46 |
| Total | 518 | 4.22 (1.08) | 518 | 3.99 (1.02) | 518 | 0.23**** | -1.81, 2.27 | 0.53 |
| Start time of eating (clock time; decimal) |  |  |  |  |  |  |  |  |
| First eating occasion | 518 | 7.86 (2.20) | 518 | 7.83 (1.83) | 518 | 0.03 | -2.82, 2.88 | 0.79 |
| Last eating occasion | 518 | 20.31 (1.63) | 518 | 19.94 (1.16) | 518 | 0.37**** | -2.40, 3.14 | 0.53 |
| First meal | 518 | 7.97 (2.40) | 518 | 7.99 (1.95) | 518 | -0.02 | -2.97, 2.93 | 0.79 |
| Last meal | 518 | 19.21 (1.22) | 518 | 19.33 (1.08) | 518 | -0.12* | -2.30, 2.06 | 0.66 |
| First snack | 422 | 14.88 (4.03) | 479 | 15.02 (3.18) | 406 | -0.25 | -7.27, 6.77 | 0.51 |
| Last snack | 422 | 18.95 (3.39) | 479 | 17.52 (2.72) | 406 | 1.28**** | -5.20, 7.76 | 0.39 |
| Breakfast | 440 | 7.07 (0.80) | 499 | 7.28 (0.93) | 438 | -0.10*** | -1.21, 1.01 | 0.79 |
| Lunch | 507 | 12.43 (0.73) | 518 | 12.61 (0.70) | 507 | -0.16**** | -1.38, 1.06 | 0.60 |
| Dinner | 512 | 19.29 (0.97) | 517 | 19.47 (0.94) | 512 | -0.17**** | -1.65, 1.31 | 0.73 |
| Duration of eating occasion (minutes; decimal) |  |  |  |  |  |  |  |  |
| First eating occasion | 518 | 15.07 (7.95) | 518 | 12.47 (5.33) | 518 | 2.60**** | -13.50, 18.70 | 0.46 |
| Last eating occasion | 518 | 15.55 (17.18) | 518 | 16.94 (7.86) | 518 | -1.39 | -37.28, 34.50 | 0.26 |
| First meal | 518 | 15.07 (7.95) | 518 | 15.43 (7.02) | 518 | -0.36 | -13.43, 12.71 | 0.57 |
| Last meal | 518 | 26.67 (14.11) | 518 | 29.87 (13.26) | 518 | -3.20**** | -28.07, 21.67 | 0.55 |
| First snack | 412 | 9.15 (11.69) | 479 | 10.24 (13.22) | 397 | -0.80 | -28.27, 26.67 | 0.32 |
| Last snack | 412 | 11.72 (14.72) | 479 | 10.89 (13.34) | 397 | 0.92 | -28.78, 30.62 | 0.42 |
| Breakfast | 441 | 14.07 (5.57) | 499 | 14.10 (6.63) | 439 | -0.16 | -11.12, 10.80 | 0.58 |
| Lunch | 508 | 18.95 (8.10) | 518 | 19.87 (7.53) | 508 | -0.91** | -14.64, 12.82 | 0.58 |
| Dinner | 512 | 26.49 (13.42) | 517 | 29.91 (13.29) | 512 | -3.43**** | -27.42, 20.56 | 0.55 |
| Eating window |  |  |  |  |  |  |  |  |
| Duration of eating window 1 (hours; decimal)^f^ | 518 | 12.45 (2.53) | 518 | 12.11 (2.01) | 518 | 0.34*** | -3.61, 4.29 | 0.59 |
| Duration of eating window 2 (hours; decimal)^g^ | 518 | 12.71 (2.51) | 518 | 12.39 (2.02) | 518 | 0.32*** | -3.69, 4.33 | 0.57 |
| Eating midpoint 1 (clock time; decimal)^f^ | 518 | 14.09 (1.46) | 518 | 13.89 (1.16) | 518 | 0.20**** | -1.80, 2.20 | 0.68 |
| Eating midpoint 2 (clock time; decimal)^g^ | 518 | 14.22 (1.47) | 518 | 14.03 (1.17) | 518 | 0.19**** | -1.84, 2.22 | 0.68 |
| Time interval between sleep and eating (hours;  decimal) |  |  |  |  |  |  |  |  |
| Wake time and first eating occasion | 518 | 1.70 (2.00) | 518 | 1.60 (1.47) | 518 | 0.10 | -2.74, 2.94 | 0.69 |
| Wake time and first meal | 518 | 1.80 (2.17) | 518 | 1.74 (1.67) | 518 | 0.06 | -2.95, 3.07 | 0.69 |
| Last eating occasion and sleep time | 518 | 3.02 (1.49) | 518 | 3.52 (1.18) | 518 | -0.50**** | -3.47, 2.47 | 0.37 |
| Last meal and sleep time | 518 | 3.89 (1.51) | 518 | 3.90 (1.24) | 518 | -0.01 | -2.66, 2.64 | 0.61 |

CNBQ, Chrono-Nutrition Behavior Questionnaire; EMA, ecological momentary assessment; SD, standard deviation.

^a^ Meals included breakfast, lunch, and dinner. All time-related variables are shown in decimal format, with the unit of *clock time; decimal* (e.g., 23.51 means 11:31 PM, while 6.18 means 6:11 AM); *hours; decimal* (e.g., 6.62 means a duration of 6 h 37 min); or *minutes; decimal* (e.g., 15.07 means a duration of 15 min 4 sec).

^b^ Based on 2–9 days’ data (median 7 days).

^c^ Calculated as the CNBQ-based value minus the EMA food diary-based value (at the individual level). Paired comparison was made using the paired t-test: * P <0.05, ** P <0.01, *** P <0.001, and **** P <0.0001.

^d^ Calculated as mean difference plus-minus 1.96 SD of the difference.

^e^ All values were significant (P <0.0001).

^f^ Calculated using the start time of first eating occasion and the start time of last eating occasion.

^g^ Calculated using the start time of first eating occasion and the finish time of last eating occasion.

Supplemental Table 6. Sleep and temporal patterns of eating variables on workdays in younger participants (aged <44 years) ^a^

|  | CNBQ | | EMA food diaries ^b^ | | Paired | Mean | Limit of | Spearman |
| --- | --- | --- | --- | --- | --- | --- | --- | --- |
|  | n | Mean (SD) | n | Mean (SD) | n | difference ^c^ | Agreement ^d^ | correlation ^e^ |
| Sleep variables |  |  |  |  |  |  |  |  |
| Sleep time (clock time; decimal) | 514 | 23.76 (1.47) | 514 | 23.66 (1.53) | 514 | 0.10 | -3.14, 3.34 | 0.68 |
| Wake time (clock time; decimal) | 514 | 6.58 (1.27) | 514 | 6.79 (1.29) | 514 | -0.21**** | -2.12, 1.70 | 0.75 |
| Daily sleep duration (hours; decimal) | 514 | 6.77 (1.13) | 514 | 6.68 (1.03) | 514 | 0.09* | -1.80, 1.98 | 0.60 |
| Mid-sleep time (clock time; decimal) | 514 | 3.20 (1.11) | 514 | 3.50 (1.33) | 514 | -0.30**** | -2.24, 1.64 | 0.76 |
| Daily eating frequency (number) |  |  |  |  |  |  |  |  |
| Meals | 514 | 2.73 (0.48) | 514 | 2.71 (0.41) | 514 | 0.02 | -0.60, 0.64 | 0.71 |
| Snacks | 514 | 1.23 (0.98) | 514 | 0.97 (0.95) | 514 | 0.25**** | -1.71, 2.21 | 0.49 |
| Total | 514 | 3.96 (1.15) | 514 | 3.68 (1.09) | 514 | 0.27**** | -1.83, 2.37 | 0.55 |
| Start time of eating (clock time; decimal) |  |  |  |  |  |  |  |  |
| First eating occasion | 514 | 8.45 (2.63) | 514 | 8.51 (2.28) | 514 | -0.06 | -3.19, 3.07 | 0.79 |
| Last eating occasion | 514 | 20.44 (1.68) | 514 | 20.14 (1.21) | 514 | 0.30**** | -2.60, 3.20 | 0.57 |
| First meal | 514 | 8.55 (2.80) | 514 | 8.65 (2.41) | 514 | -0.10 | -3.11, 2.91 | 0.80 |
| Last meal | 514 | 19.39 (1.26) | 514 | 19.55 (1.12) | 514 | -0.16** | -2.40, 2.08 | 0.66 |
| First snack | 375 | 15.58 (4.41) | 439 | 15.86 (3.44) | 344 | -0.15 | -8.48, 8.18 | 0.43 |
| Last snack | 375 | 19.45 (3.43) | 439 | 18.18 (2.88) | 344 | 1.27**** | -5.36, 7.90 | 0.37 |
| Breakfast | 393 | 7.17 (0.95) | 473 | 7.45 (1.08) | 389 | -0.12*** | -1.50, 1.26 | 0.72 |
| Lunch | 500 | 12.48 (0.74) | 512 | 12.65 (0.72) | 499 | -0.16**** | -1.43, 1.11 | 0.60 |
| Dinner | 510 | 19.44 (1.10) | 514 | 19.68 (1.03) | 510 | -0.23**** | -1.95, 1.49 | 0.69 |
| Duration of eating occasion (minutes; decimal) |  |  |  |  |  |  |  |  |
| First eating occasion | 514 | 13.91 (6.45) | 514 | 11.30 (4.89) | 514 | 2.61**** | -9.61, 14.83 | 0.45 |
| Last eating occasion | 514 | 17.23 (19.91) | 514 | 18.27 (8.60) | 514 | -1.04 | -40.95, 38.87 | 0.27 |
| First meal | 514 | 13.91 (6.45) | 514 | 13.86 (6.54) | 514 | 0.05 | -11.81, 11.91 | 0.53 |
| Last meal | 514 | 25.67 (15.19) | 514 | 28.39 (13.25) | 514 | -2.72**** | -30.73, 25.29 | 0.52 |
| First snack | 366 | 10.13 (14.28) | 439 | 12.56 (20.62) | 336 | -2.00 | -40.57, 36.57 | 0.38 |
| Last snack | 366 | 13.32 (18.80) | 439 | 14.17 (22.38) | 336 | -0.66 | -45.05, 43.73 | 0.43 |
| Breakfast | 395 | 12.89 (5.83) | 473 | 11.99 (6.05) | 391 | 0.53 | -10.55, 11.61 | 0.54 |
| Lunch | 501 | 17.50 (6.59) | 512 | 17.89 (7.18) | 500 | -0.32 | -12.14, 11.50 | 0.59 |
| Dinner | 510 | 25.71 (15.23) | 514 | 28.45 (13.33) | 510 | -2.69**** | -30.78, 25.40 | 0.52 |
| Eating window |  |  |  |  |  |  |  |  |
| Duration of eating window 1 (hours; decimal)^f^ | 514 | 12.00 (2.87) | 514 | 11.63 (2.39) | 514 | 0.36**** | -3.76, 4.48 | 0.66 |
| Duration of eating window 2 (hours; decimal)^g^ | 514 | 12.29 (2.85) | 514 | 11.94 (2.40) | 514 | 0.35*** | -3.86, 4.56 | 0.64 |
| Eating midpoint 1 (clock time; decimal)^f^ | 514 | 14.45 (1.67) | 514 | 14.32 (1.37) | 514 | 0.12* | -2.08, 2.32 | 0.73 |
| Eating midpoint 2 (clock time; decimal)^g^ | 514 | 14.59 (1.70) | 514 | 14.48 (1.39) | 514 | 0.11* | -2.11, 2.33 | 0.73 |
| Time interval between sleep and eating (hours;  decimal) |  |  |  |  |  |  |  |  |
| Wake time and first eating occasion | 514 | 1.90 (2.42) | 514 | 1.82 (1.94) | 514 | 0.08 | -2.95, 3.11 | 0.74 |
| Wake time and first meal | 514 | 2.00 (2.58) | 514 | 1.92 (2.07) | 514 | 0.08 | -2.87, 3.03 | 0.75 |
| Last eating occasion and sleep time | 514 | 3.09 (1.63) | 514 | 3.46 (1.26) | 514 | -0.37**** | -3.57, 2.83 | 0.41 |
| Last meal and sleep time | 514 | 3.98 (1.63) | 514 | 3.86 (1.37) | 514 | 0.12* | -2.60, 2.84 | 0.64 |

CNBQ, Chrono-Nutrition Behavior Questionnaire; EMA, ecological momentary assessment; SD, standard deviation.

^a^ Meals included breakfast, lunch, and dinner. All time-related variables are shown in decimal format, with the unit of *clock time; decimal* (e.g., 23.76 means 11:46 PM, while 6.58 means 6:35 AM); *hours; decimal* (e.g., 6.77 means a duration of 6 h 46 min); or *minutes; decimal* (e.g., 13.91 means a duration of 13 min 55 sec).

^b^ Based on 2–9 days’ data (median 4 days).

^c^ Calculated as the CNBQ-based value minus the EMA food diary-based value (at the individual level). Paired comparison was made using the paired t-test: * P <0.05, ** P <0.01, *** P <0.001, and **** P <0.0001.

^d^ Calculated as mean difference plus-minus 1.96 SD of the difference.

^e^ All values were significant (P <0.0001).

^f^ Calculated using the start time of first eating occasion and the start time of last eating occasion.

^g^ Calculated using the start time of first eating occasion and the finish time of last eating occasion.

Supplemental Table 7. Sleep and temporal patterns of eating variables on workdays in older participants (aged ≥44 years) ^a^

|  | CNBQ | | EMA food diaries ^b^ | | Paired | Mean | Limit of | Spearman |
| --- | --- | --- | --- | --- | --- | --- | --- | --- |
|  | n | Mean (SD) | n | Mean (SD) | n | difference ^c^ | Agreement ^d^ | correlation ^e^ |
| Sleep variables |  |  |  |  |  |  |  |  |
| Sleep time (clock time; decimal) | 536 | 23.27 (1.66) | 536 | 23.46 (1.21) | 536 | -0.19** | -3.14, 2.76 | 0.76 |
| Wake time (clock time; decimal) | 536 | 5.91 (0.97) | 536 | 5.98 (1.00) | 536 | -0.07** | -1.33, 1.19 | 0.77 |
| Daily sleep duration (hours; decimal) | 536 | 6.55 (1.18) | 536 | 6.38 (1.01) | 536 | 0.16**** | -1.61, 1.93 | 0.66 |
| Mid-sleep time (clock time; decimal) | 536 | 2.86 (2.22) | 536 | 2.99 (1.51) | 536 | -0.13 | -3.25, 2.99 | 0.79 |
| Daily eating frequency (number) |  |  |  |  |  |  |  |  |
| Meals | 536 | 2.83 (0.41) | 536 | 2.83 (0.36) | 536 | 0.00 | -0.51, 0.51 | 0.66 |
| Snacks | 536 | 1.18 (0.98) | 536 | 1.01 (0.85) | 536 | 0.18**** | -1.71, 2.07 | 0.51 |
| Total | 536 | 4.02 (1.11) | 536 | 3.84 (0.95) | 536 | 0.18**** | -1.80, 2.16 | 0.56 |
| Start time of eating (clock time; decimal) |  |  |  |  |  |  |  |  |
| First eating occasion | 536 | 7.68 (2.19) | 536 | 7.65 (1.98) | 536 | 0.03 | -2.60, 2.66 | 0.81 |
| Last eating occasion | 536 | 20.29 (1.76) | 536 | 20.05 (1.28) | 536 | 0.24*** | -2.57, 3.05 | 0.60 |
| First meal | 536 | 7.75 (2.35) | 536 | 7.82 (2.12) | 536 | -0.07 | -2.83, 2.69 | 0.80 |
| Last meal | 536 | 19.41 (1.42) | 536 | 19.52 (1.21) | 536 | -0.11* | -2.10, 1.88 | 0.73 |
| First snack | 380 | 15.42 (4.36) | 468 | 15.50 (3.79) | 366 | -0.19 | -8.14, 7.76 | 0.51 |
| Last snack | 380 | 19.24 (3.27) | 468 | 17.81 (3.14) | 366 | 1.30**** | -5.43, 8.03 | 0.40 |
| Breakfast | 464 | 6.94 (0.82) | 499 | 7.09 (0.88) | 463 | -0.10**** | -1.18, 0.98 | 0.80 |
| Lunch | 525 | 12.41 (0.88) | 533 | 12.53 (0.74) | 525 | -0.11** | -1.56, 1.34 | 0.62 |
| Dinner | 530 | 19.49 (1.19) | 535 | 19.60 (1.11) | 530 | -0.11*** | -1.54, 1.32 | 0.77 |
| Duration of eating occasion (minutes; decimal) |  |  |  |  |  |  |  |  |
| First eating occasion | 536 | 14.57 (8.16) | 536 | 11.74 (5.09) | 536 | 2.83**** | -13.60, 19.26 | 0.46 |
| Last eating occasion | 536 | 17.70 (17.87) | 536 | 18.35 (9.93) | 536 | -0.65 | -38.14, 36.84 | 0.29 |
| First meal | 536 | 14.57 (8.16) | 536 | 15.13 (7.62) | 536 | -0.56 | -15.26, 14.14 | 0.62 |
| Last meal | 536 | 28.00 (17.06) | 536 | 32.47 (19.20) | 536 | -4.47**** | -34.84, 25.90 | 0.63 |
| First snack | 370 | 9.66 (9.07) | 468 | 11.37 (15.78) | 359 | -0.92 | -22.03, 20.19 | 0.42 |
| Last snack | 370 | 11.49 (11.56) | 468 | 11.95 (15.78) | 359 | 0.19 | -21.50, 21.88 | 0.43 |
| Breakfast | 466 | 14.02 (6.35) | 499 | 14.10 (6.35) | 465 | -0.11 | -11.34, 11.12 | 0.64 |
| Lunch | 527 | 17.27 (7.81) | 533 | 18.50 (7.62) | 527 | -1.10*** | -14.13, 11.93 | 0.64 |
| Dinner | 529 | 27.94 (16.53) | 535 | 32.43 (19.18) | 529 | -4.56**** | -34.08, 24.96 | 0.64 |
| Eating window |  |  |  |  |  |  |  |  |
| Duration of eating window 1 (hours; decimal)^f^ | 536 | 12.61 (2.66) | 536 | 12.40 (2.18) | 536 | 0.21* | -3.73, 4.15 | 0.65 |
| Duration of eating window 2 (hours; decimal)^g^ | 536 | 12.90 (2.63) | 536 | 12.71 (2.19) | 536 | 0.20* | -3.77, 4.17 | 0.63 |
| Eating midpoint 1 (clock time; decimal)^f^ | 536 | 13.99 (1.48) | 536 | 13.85 (1.26) | 536 | 0.14** | -1.74, 2.02 | 0.74 |
| Eating midpoint 2 (clock time; decimal)^g^ | 536 | 14.13 (1.48) | 536 | 14.00 (1.28) | 536 | 0.13** | -1.78, 2.04 | 0.73 |
| Time interval between sleep and eating (hours;  decimal) |  |  |  |  |  |  |  |  |
| Wake time and first eating occasion | 536 | 1.79 (2.21) | 536 | 1.72 (1.97) | 536 | 0.06 | -2.58, 2.70 | 0.72 |
| Wake time and first meal | 536 | 1.85 (2.34) | 536 | 1.87 (2.13) | 536 | -0.02 | -2.84, 2.80 | 0.70 |
| Last eating occasion and sleep time | 536 | 2.82 (1.54) | 536 | 3.20 (1.15) | 536 | -0.39**** | -3.22, 2.44 | 0.47 |
| Last meal and sleep time | 536 | 3.47 (1.50) | 536 | 3.48 (1.24) | 536 | -0.01 | -2.43, 2.41 | 0.68 |

CNBQ, Chrono-Nutrition Behavior Questionnaire; EMA, ecological momentary assessment; SD, standard deviation.

^a^ Meals included breakfast, lunch, and dinner. All time-related variables are shown in decimal format, with the unit of *clock time; decimal* (e.g., 23.27 means 11:16 PM, while 5.91 means 5:55 AM); *hours; decimal* (e.g., 6.55 means a duration of 6 h 33 min); or *minutes; decimal* (e.g., 14.57 means a duration of 14 min 34 sec).

^b^ Based on 2–9 days’ data (median 4 days).

^c^ Calculated as the CNBQ-based value minus the EMA food diary-based value (at the individual level). Paired comparison was made using the paired t-test: * P <0.05, ** P <0.01, *** P <0.001, and **** P <0.0001.

^d^ Calculated as mean difference plus-minus 1.96 SD of the difference.

^e^ All values were significant (P <0.0001).

^f^ Calculated using the start time of first eating occasion and the start time of last eating occasion.

^g^ Calculated using the start time of first eating occasion and the finish time of last eating occasion.

Supplemental Table 8. Sleep and temporal patterns of eating variables on workdays in shift working participants ^a^

|  | CNBQ | | EMA food diaries ^b^ | | Paired | Mean | Limit of | Spearman |
| --- | --- | --- | --- | --- | --- | --- | --- | --- |
|  | n | Mean (SD) | n | Mean (SD) | n | difference ^c^ | Agreement ^d^ | correlation ^e^ |
| Sleep variables |  |  |  |  |  |  |  |  |
| Sleep time (clock time; decimal) | 246 | 23.58 (2.46) | 246 | 23.54 (1.72) | 246 | 0.04 | -5.58, 5.66 | 0.48 |
| Wake time (clock time; decimal) | 246 | 6.49 (1.53) | 246 | 6.91 (1.65) | 246 | -0.42**** | -3.20, 2.36 | 0.56 |
| Daily sleep duration (hours; decimal) | 246 | 6.62 (1.41) | 246 | 6.38 (1.11) | 246 | 0.24** | -2.18, 2.66 | 0.51 |
| Mid-sleep time (clock time; decimal) | 246 | 3.28 (1.83) | 246 | 3.91 (1.80) | 246 | -0.63**** | -3.64, 2.38 | 0.60 |
| Daily eating frequency (number) |  |  |  |  |  |  |  |  |
| Meals | 246 | 2.65 (0.53) | 246 | 2.62 (0.44) | 246 | 0.03 | -0.71, 0.77 | 0.67 |
| Snacks | 246 | 1.10 (0.95) | 246 | 0.79 (0.77) | 246 | 0.30**** | -1.65, 2.25 | 0.34 |
| Total | 246 | 3.75 (1.16) | 246 | 3.42 (0.94) | 246 | 0.33**** | -1.84, 2.50 | 0.46 |
| Start time of eating (clock time; decimal) |  |  |  |  |  |  |  |  |
| First eating occasion | 246 | 8.80 (3.07) | 246 | 8.72 (2.40) | 246 | 0.08 | -3.85, 4.01 | 0.69 |
| Last eating occasion | 246 | 20.71 (2.15) | 246 | 20.35 (1.50) | 246 | 0.36** | -3.36, 4.08 | 0.48 |
| First meal | 246 | 8.90 (3.20) | 246 | 9.05 (2.54) | 246 | -0.15 | -4.01, 3.71 | 0.71 |
| Last meal | 246 | 19.55 (1.77) | 246 | 19.74 (1.46) | 246 | -0.19 | -3.35, 2.97 | 0.52 |
| First snack | 169 | 16.62 (4.72) | 205 | 16.11 (4.01) | 149 | 0.73 | -10.10, 11.56 | 0.24 |
| Last snack | 169 | 20.19 (3.49) | 205 | 18.23 (3.35) | 149 | 2.28**** | -6.04, 10.60 | 0.19 |
| Breakfast | 174 | 7.10 (1.15) | 225 | 7.52 (1.18) | 174 | -0.20* | -2.32, 1.92 | 0.47 |
| Lunch | 237 | 12.58 (1.04) | 246 | 12.79 (1.00) | 237 | -0.19** | -1.93, 1.55 | 0.49 |
| Dinner | 241 | 19.70 (1.42) | 245 | 19.92 (1.29) | 241 | -0.20** | -2.47, 2.07 | 0.56 |
| Duration of eating occasion (minutes; decimal) |  |  |  |  |  |  |  |  |
| First eating occasion | 246 | 15.29 (10.56) | 246 | 11.68 (4.71) | 246 | 3.60**** | -17.63, 24.83 | 0.33 |
| Last eating occasion | 246 | 18.87 (20.81) | 246 | 18.97 (7.45) | 246 | -0.10 | -40.41, 40.21 | 0.17 |
| First meal | 246 | 15.29 (10.56) | 246 | 15.63 (7.16) | 246 | -0.34 | -16.92, 16.24 | 0.46 |
| Last meal | 246 | 27.35 (18.38) | 246 | 29.34 (14.09) | 246 | -1.99* | -31.42, 27.44 | 0.55 |
| First snack | 165 | 10.72 (9.81) | 205 | 13.11 (16.67) | 146 | -1.27 | -25.48, 22.94 | 0.36 |
| Last snack | 165 | 13.86 (16.99) | 205 | 14.87 (21.12) | 146 | 0.05 | -39.25, 39.35 | 0.35 |
| Breakfast | 177 | 13.77 (7.36) | 225 | 13.18 (6.00) | 177 | 0.05 | -14.06, 14.16 | 0.47 |
| Lunch | 238 | 17.91 (9.94) | 246 | 18.86 (7.72) | 238 | -0.86 | -16.26, 14.54 | 0.55 |
| Dinner | 240 | 27.05 (17.32) | 245 | 29.34 (14.10) | 240 | -2.41** | -30.12, 25.30 | 0.56 |
| Eating window |  |  |  |  |  |  |  |  |
| Duration of eating window 1 (hours; decimal)^f^ | 246 | 11.91 (3.47) | 246 | 11.63 (2.54) | 246 | 0.28 | -5.13, 5.69 | 0.58 |
| Duration of eating window 2 (hours; decimal)^g^ | 246 | 12.22 (3.40) | 246 | 11.95 (2.54) | 246 | 0.28 | -5.12, 5.68 | 0.56 |
| Eating midpoint 1 (clock time; decimal)^f^ | 246 | 14.76 (2.00) | 246 | 14.53 (1.55) | 246 | 0.22* | -2.48, 2.92 | 0.70 |
| Eating midpoint 2 (clock time; decimal)^g^ | 246 | 14.91 (2.02) | 246 | 14.69 (1.57) | 246 | 0.22* | -2.51, 2.95 | 0.70 |
| Time interval between sleep and eating (hours;  decimal) |  |  |  |  |  |  |  |  |
| Wake time and first eating occasion | 246 | 2.38 (2.88) | 246 | 2.11 (2.05) | 246 | 0.27* | -3.42, 3.96 | 0.70 |
| Wake time and first meal | 246 | 2.47 (3.02) | 246 | 2.33 (2.22) | 246 | 0.13 | -3.58, 3.84 | 0.70 |
| Last eating occasion and sleep time | 246 | 2.85 (2.00) | 246 | 3.33 (1.38) | 246 | -0.48*** | -4.34, 3.38 | 0.38 |
| Last meal and sleep time | 246 | 3.81 (2.04) | 246 | 3.74 (1.46) | 246 | 0.07 | -3.72, 3.86 | 0.52 |

CNBQ, Chrono-Nutrition Behavior Questionnaire; EMA, ecological momentary assessment; SD, standard deviation.

^a^ Meals included breakfast, lunch, and dinner. All time-related variables are shown in decimal format, with the unit of *clock time; decimal* (e.g., 23.58 means 11:35 PM, while 6.49 means 6:29 AM); *hours; decimal* (e.g., 6.62 means a duration of 6 h 37 min); or *minutes; decimal* (e.g., 15.29 means a duration of 15 min 17 sec).

^b^ Based on 2–9 days’ data (median 7 days).

^c^ Calculated as the CNBQ-based value minus the EMA food diary-based value (at the individual level). Paired comparison was made using the paired t-test: * P <0.05, ** P <0.01, *** P <0.001, and **** P <0.0001.

^d^ Calculated as mean difference plus-minus 1.96 SD of the difference.

^e^ All values were significant: P = 0.003 for start time of first snack, 0.02 for start time of last snack, 0.007 for time spent on last eating occasion, and <0.0001 for all other variables.

^f^ Calculated using the start time of first eating occasion and the start time of last eating occasion.

^g^ Calculated using the start time of first eating occasion and the finish time of last eating occasion.

Supplemental Table 9. Sleep and temporal patterns of eating variables on workdays in non-shift working participants ^a^

|  | CNBQ | | EMA food diaries ^b^ | | Paired | Mean | Limit of | Spearman |
| --- | --- | --- | --- | --- | --- | --- | --- | --- |
|  | n | Mean (SD) | n | Mean (SD) | n | difference ^c^ | Agreement ^d^ | correlation ^e^ |
| Sleep variables |  |  |  |  |  |  |  |  |
| Sleep time (clock time; decimal) | 804 | 23.49 (1.20) | 804 | 23.57 (1.25) | 804 | -0.08* | -1.80, 1.64 | 0.80 |
| Wake time (clock time; decimal) | 804 | 6.16 (1.03) | 804 | 6.21 (1.00) | 804 | -0.06** | -1.02, 0.90 | 0.88 |
| Daily sleep duration (hours; decimal) | 804 | 6.67 (1.08) | 804 | 6.58 (1.00) | 804 | 0.09** | -1.52, 1.70 | 0.69 |
| Mid-sleep time (clock time; decimal) | 804 | 2.94 (1.75) | 804 | 3.03 (1.25) | 804 | -0.09* | -2.51, 2.33 | 0.85 |
| Daily eating frequency (number) |  |  |  |  |  |  |  |  |
| Meals | 804 | 2.82 (0.41) | 804 | 2.82 (0.36) | 804 | 0.01 | -0.50, 0.52 | 0.69 |
| Snacks | 804 | 1.24 (0.99) | 804 | 1.05 (0.92) | 804 | 0.19**** | -1.73, 2.11 | 0.54 |
| Total | 804 | 4.06 (1.11) | 804 | 3.87 (1.02) | 804 | 0.19**** | -1.81, 2.19 | 0.57 |
| Start time of eating (clock time; decimal) |  |  |  |  |  |  |  |  |
| First eating occasion | 804 | 7.83 (2.17) | 804 | 7.87 (2.06) | 804 | -0.04 | -2.52, 2.44 | 0.86 |
| Last eating occasion | 804 | 20.26 (1.55) | 804 | 20.02 (1.14) | 804 | 0.24**** | -2.29, 2.77 | 0.62 |
| First meal | 804 | 7.91 (2.35) | 804 | 7.98 (2.17) | 804 | -0.07 | -2.58, 2.44 | 0.86 |
| Last meal | 804 | 19.35 (1.17) | 804 | 19.47 (1.05) | 804 | -0.12*** | -1.79, 1.55 | 0.77 |
| First snack | 586 | 15.18 (4.23) | 702 | 15.55 (3.50) | 561 | -0.41** | -7.60, 6.78 | 0.54 |
| Last snack | 586 | 19.10 (3.28) | 702 | 17.92 (2.92) | 561 | 1.02**** | -5.05, 7.09 | 0.45 |
| Breakfast | 683 | 7.03 (0.81) | 747 | 7.19 (0.93) | 678 | -0.09**** | -0.94, 0.76 | 0.86 |
| Lunch | 788 | 12.40 (0.73) | 799 | 12.53 (0.62) | 787 | -0.11**** | -1.34, 1.12 | 0.65 |
| Dinner | 799 | 19.40 (1.04) | 804 | 19.56 (0.98) | 799 | -0.16**** | -1.47, 1.15 | 0.80 |
| Duration of eating occasion (minutes; decimal) |  |  |  |  |  |  |  |  |
| First eating occasion | 804 | 13.93 (6.06) | 804 | 11.48 (5.08) | 804 | 2.45**** | -9.24, 14.14 | 0.49 |
| Last eating occasion | 804 | 17.04 (18.26) | 804 | 18.11 (9.79) | 804 | -1.07 | -39.25, 37.11 | 0.30 |
| First meal | 804 | 13.93 (6.06) | 804 | 14.16 (7.10) | 804 | -0.23 | -12.49, 12.03 | 0.62 |
| Last meal | 804 | 26.71 (15.49) | 804 | 30.82 (17.38) | 804 | -4.11**** | -33.29, 25.07 | 0.58 |
| First snack | 571 | 9.66 (12.49) | 702 | 11.61 (18.72) | 549 | -1.49* | -33.85, 30.87 | 0.40 |
| Last snack | 571 | 11.98 (15.17) | 702 | 12.48 (18.69) | 549 | -0.29 | -33.52, 32.94 | 0.45 |
| Breakfast | 684 | 13.43 (5.78) | 747 | 13.04 (6.38) | 679 | 0.22 | -10.06, 10.50 | 0.64 |
| Lunch | 790 | 17.22 (6.20) | 799 | 18.00 (7.31) | 789 | -0.67** | -12.13, 10.79 | 0.64 |
| Dinner | 799 | 26.78 (15.51) | 804 | 30.83 (17.39) | 799 | -4.02**** | -33.21, 25.17 | 0.59 |
| Eating window |  |  |  |  |  |  |  |  |
| Duration of eating window 1 (hours; decimal)^f^ | 804 | 12.43 (2.52) | 804 | 12.15 (2.23) | 804 | 0.29**** | -3.21, 3.79 | 0.70 |
| Duration of eating window 2 (hours; decimal)^g^ | 804 | 12.72 (2.52) | 804 | 12.45 (2.24) | 804 | 0.27**** | -3.33, 3.87 | 0.68 |
| Eating midpoint 1 (clock time; decimal)^f^ | 804 | 14.04 (1.41) | 804 | 13.94 (1.24) | 804 | 0.10** | -1.69, 1.89 | 0.75 |
| Eating midpoint 2 (clock time; decimal)^g^ | 804 | 14.19 (1.42) | 804 | 14.09 (1.26) | 804 | 0.09** | -1.73, 1.91 | 0.74 |
| Time interval between sleep and eating (hours;  decimal) |  |  |  |  |  |  |  |  |
| Wake time and first eating occasion | 804 | 1.68 (2.09) | 804 | 1.66 (1.91) | 804 | 0.01 | -2.50, 2.52 | 0.75 |
| Wake time and first meal | 804 | 1.76 (2.24) | 804 | 1.76 (2.05) | 804 | -0.01 | -2.59, 2.57 | 0.75 |
| Last eating occasion and sleep time | 804 | 2.98 (1.44) | 804 | 3.33 (1.16) | 804 | -0.35**** | -3.05, 2.35 | 0.47 |
| Last meal and sleep time | 804 | 3.69 (1.42) | 804 | 3.65 (1.27) | 804 | 0.05 | -2.02, 2.12 | 0.71 |

CNBQ, Chrono-Nutrition Behavior Questionnaire; EMA, ecological momentary assessment; SD, standard deviation.

^a^ Meals included breakfast, lunch, and dinner. All time-related variables are shown in decimal format, with the unit of *clock time; decimal* (e.g., 23.49 means 11:29 PM, while 6.16 means 6:10 AM); *hours; decimal* (e.g., 6.67 means a duration of 6 h 40 min); or *minutes; decimal* (e.g., 13.93 means a duration of 13 min 56 sec).

^b^ Based on 2–9 days’ data (median 7 days).

^c^ Calculated as the CNBQ-based value minus the EMA food diary-based value (at the individual level). Paired comparison was made using the paired t-test: * P <0.05, ** P <0.01, *** P <0.001, and **** P <0.0001.

^d^ Calculated as mean difference plus-minus 1.96 SD of the difference.

^e^ All values were significant (P <0.0001).

^f^ Calculated using the start time of first eating occasion and the start time of last eating occasion.

^g^ Calculated using the start time of first eating occasion and the finish time of last eating occasion.

Supplemental Table 10. Sleep and temporal patterns of eating variables on non-workdays in male participants ^a^

|  | CNBQ | | EMA food diaries ^b^ | | Paired | Mean | Limit of | Spearman |
| --- | --- | --- | --- | --- | --- | --- | --- | --- |
|  | n | Mean (SD) | n | Mean (SD) | n | difference ^c^ | Agreement ^d^ | correlation ^e^ |
| Sleep variables |  |  |  |  |  |  |  |  |
| Sleep time (clock time; decimal) | 532 | 23.44 (3.27) | 532 | 23.94 (1.68) | 532 | -0.50*** | -6.84, 5.84 | 0.65 |
| Wake time (clock time; decimal) | 532 | 7.51 (1.65) | 532 | 7.49 (1.61) | 532 | 0.02 | -2.55, 2.59 | 0.71 |
| Daily sleep duration (hours; decimal) | 532 | 7.71 (1.45) | 532 | 7.27 (1.25) | 532 | 0.44**** | -2.42, 3.30 | 0.45 |
| Mid-sleep time (clock time; decimal) | 532 | 3.75 (1.86) | 532 | 3.90 (1.51) | 532 | -0.15* | -3.54, 3.24 | 0.74 |
| Daily eating frequency (number) |  |  |  |  |  |  |  |  |
| Meals | 532 | 2.70 (0.49) | 532 | 2.67 (0.45) | 532 | 0.04* | -0.72, 0.80 | 0.61 |
| Snacks | 532 | 1.09 (1.00) | 532 | 0.86 (0.76) | 532 | 0.22**** | -1.70, 2.14 | 0.45 |
| Total | 532 | 3.79 (1.13) | 532 | 3.53 (0.95) | 532 | 0.26**** | -1.87, 2.39 | 0.49 |
| Start time of eating (clock time; decimal) |  |  |  |  |  |  |  |  |
| First eating occasion | 532 | 9.22 (2.28) | 532 | 9.24 (2.14) | 532 | -0.01 | -3.21, 3.19 | 0.74 |
| Last eating occasion | 532 | 19.89 (1.68) | 532 | 19.65 (1.22) | 532 | 0.24*** | -2.77, 3.25 | 0.50 |
| First meal | 532 | 9.33 (2.42) | 532 | 9.36 (2.22) | 532 | -0.03 | -3.34, 3.28 | 0.75 |
| Last meal | 532 | 18.87 (1.06) | 532 | 19.08 (0.99) | 532 | -0.21**** | -2.18, 1.76 | 0.62 |
| First snack | 339 | 15.32 (4.10) | 449 | 15.30 (3.20) | 317 | -0.06 | -8.05, 7.93 | 0.39 |
| Last snack | 339 | 19.40 (3.03) | 449 | 17.46 (3.04) | 317 | 1.79**** | -5.14, 8.72 | 0.28 |
| Breakfast | 393 | 8.09 (1.14) | 479 | 8.29 (1.08) | 389 | -0.05 | -1.95, 1.85 | 0.69 |
| Lunch | 518 | 12.54 (0.76) | 524 | 12.71 (0.74) | 513 | -0.18**** | -1.66, 1.30 | 0.53 |
| Dinner | 527 | 18.92 (0.93) | 532 | 19.20 (0.90) | 527 | -0.28**** | -1.77, 1.21 | 0.67 |
| Duration of eating occasion (minutes; decimal) |  |  |  |  |  |  |  |  |
| First eating occasion | 532 | 16.52 (8.87) | 532 | 13.63 (6.42) | 532 | 2.89**** | -15.04, 20.82 | 0.39 |
| Last eating occasion | 532 | 21.37 (22.80) | 532 | 20.79 (12.06) | 532 | 0.57 | -48.27, 49.41 | 0.18 |
| First meal | 532 | 16.52 (8.87) | 532 | 17.97 (10.92) | 532 | -1.45*** | -20.85, 17.95 | 0.54 |
| Last meal | 532 | 29.77 (20.65) | 532 | 36.75 (22.86) | 532 | -6.99**** | -42.18, 28.20 | 0.57 |
| First snack | 340 | 12.01 (11.53) | 449 | 15.62 (21.00) | 319 | -4.00**** | -38.91, 30.91 | 0.39 |
| Last snack | 340 | 14.96 (18.11) | 449 | 17.97 (27.56) | 319 | -3.76** | -48.37, 40.85 | 0.49 |
| Breakfast | 398 | 14.75 (6.69) | 479 | 15.60 (8.70) | 391 | -1.35*** | -15.19, 12.49 | 0.56 |
| Lunch | 521 | 19.84 (9.42) | 524 | 21.97 (9.25) | 515 | -2.12**** | -20.83, 16.59 | 0.46 |
| Dinner | 528 | 29.89 (20.68) | 532 | 36.62 (22.91) | 528 | -6.79**** | -42.10, 28.52 | 0.58 |
| Eating window |  |  |  |  |  |  |  |  |
| Duration of eating window 1 (hours; decimal)^f^ | 532 | 10.67 (2.55) | 532 | 10.42 (2.21) | 532 | 0.26** | -4.19, 4.71 | 0.52 |
| Duration of eating window 2 (hours; decimal)^g^ | 532 | 11.03 (2.49) | 532 | 10.76 (2.23) | 532 | 0.27** | -4.26, 4.80 | 0.49 |
| Eating midpoint 1 (clock time; decimal)^f^ | 532 | 14.56 (1.54) | 532 | 14.44 (1.34) | 532 | 0.11* | -2.06, 2.28 | 0.73 |
| Eating midpoint 2 (clock time; decimal)^g^ | 532 | 14.73 (1.56) | 532 | 14.62 (1.37) | 532 | 0.12* | -2.09, 2.33 | 0.73 |
| Time interval between sleep and eating (hours;  decimal) |  |  |  |  |  |  |  |  |
| Wake time and first eating occasion | 532 | 1.73 (1.87) | 532 | 1.79 (1.63) | 532 | -0.07 | -3.25, 3.11 | 0.55 |
| Wake time and first meal | 532 | 1.83 (1.99) | 532 | 1.90 (1.73) | 532 | -0.07 | -3.44, 3.30 | 0.56 |
| Last eating occasion and sleep time | 532 | 3.50 (1.72) | 532 | 4.10 (1.42) | 532 | -0.60**** | -4.08, 2.88 | 0.38 |
| Last meal and sleep time | 532 | 4.36 (1.70) | 532 | 4.39 (1.50) | 532 | -0.03 | -2.96, 2.90 | 0.59 |

CNBQ, Chrono-Nutrition Behavior Questionnaire; EMA, ecological momentary assessment; SD, standard deviation.

^a^ Meals included breakfast, lunch, and dinner. All time-related variables are shown in decimal format, with the unit of *clock time; decimal* (e.g., 23.44 means 11:26 PM, while 7.51 means 7:31 AM); *hours; decimal* (e.g., 7.71 means a duration of 7 h 43 min); or *minutes; decimal* (e.g., 16.52 means a duration of 16 min 31 sec).

^b^ Based on 2–9 days’ data (median 4 days).

^c^ Calculated as the CNBQ-based value minus the EMA food diary-based value (at the individual level). Paired comparison was made using the paired t-test: * P <0.05, ** P <0.01, *** P <0.001, and **** P <0.0001.

^d^ Calculated as mean difference plus-minus 1.96 SD of the difference.

^e^ All values were significant (P <0.0001).

^f^ Calculated using the start time of first eating occasion and the start time of last eating occasion.

^g^ Calculated using the start time of first eating occasion and the finish time of last eating occasion.

Supplemental Table 11. Sleep and temporal patterns of eating variables on non-workdays in female participants ^a^

|  | CNBQ | | EMA food diaries ^b^ | | Paired | Mean | Limit of | Spearman |
| --- | --- | --- | --- | --- | --- | --- | --- | --- |
|  | n | Mean (SD) | n | Mean (SD) | n | difference ^c^ | Agreement ^d^ | correlation ^e^ |
| Sleep variables |  |  |  |  |  |  |  |  |
| Sleep time (clock time; decimal) | 518 | 23.80 (1.66) | 518 | 23.93 (1.67) | 518 | -0.13 | -3.83, 3.57 | 0.65 |
| Wake time (clock time; decimal) | 518 | 7.41 (1.54) | 518 | 7.23 (1.37) | 518 | 0.18**** | -1.68, 2.04 | 0.77 |
| Daily sleep duration (hours; decimal) | 518 | 7.56 (1.39) | 518 | 7.07 (1.23) | 518 | 0.49**** | -2.09, 3.07 | 0.50 |
| Mid-sleep time (clock time; decimal) | 518 | 3.63 (1.24) | 518 | 3.73 (1.25) | 518 | -0.10** | -1.66, 1.46 | 0.81 |
| Daily eating frequency (number) |  |  |  |  |  |  |  |  |
| Meals | 518 | 2.75 (0.48) | 518 | 2.72 (0.38) | 518 | 0.02 | -0.75, 0.79 | 0.56 |
| Snacks | 518 | 1.44 (1.00) | 518 | 1.25 (0.83) | 518 | 0.18**** | -1.77, 2.13 | 0.45 |
| Total | 518 | 4.18 (1.17) | 518 | 3.98 (0.98) | 518 | 0.21**** | -1.94, 2.36 | 0.50 |
| Start time of eating (clock time; decimal) |  |  |  |  |  |  |  |  |
| First eating occasion | 518 | 9.03 (2.12) | 518 | 8.89 (1.77) | 518 | 0.14* | -2.54, 2.82 | 0.78 |
| Last eating occasion | 518 | 19.72 (1.55) | 518 | 19.56 (1.15) | 518 | 0.15* | -2.58, 2.88 | 0.52 |
| First meal | 518 | 9.18 (2.37) | 518 | 9.02 (1.87) | 518 | 0.16* | -2.85, 3.17 | 0.77 |
| Last meal | 518 | 18.74 (0.97) | 518 | 18.88 (1.04) | 518 | -0.14*** | -1.89, 1.61 | 0.60 |
| First snack | 413 | 13.95 (3.31) | 500 | 14.48 (2.65) | 409 | -0.37* | -7.48, 6.74 | 0.25 |
| Last snack | 413 | 18.31 (3.22) | 500 | 17.08 (2.50) | 409 | 1.15**** | -5.30, 7.60 | 0.39 |
| Breakfast | 410 | 8.17 (1.10) | 482 | 8.27 (1.02) | 407 | 0.04 | -1.43, 1.51 | 0.74 |
| Lunch | 498 | 12.57 (0.77) | 516 | 12.77 (0.71) | 497 | -0.20**** | -1.76, 1.36 | 0.45 |
| Dinner | 514 | 18.78 (0.84) | 516 | 19.08 (0.83) | 514 | -0.30**** | -1.62, 1.02 | 0.66 |
| Duration of eating occasion (minutes; decimal) |  |  |  |  |  |  |  |  |
| First eating occasion | 518 | 19.78 (11.51) | 518 | 16.50 (7.90) | 518 | 3.28**** | -20.63, 27.19 | 0.34 |
| Last eating occasion | 518 | 18.39 (17.83) | 518 | 20.42 (11.38) | 518 | -2.03* | -39.92, 35.86 | 0.23 |
| First meal | 518 | 19.78 (11.51) | 518 | 21.05 (14.64) | 518 | -1.27 | -30.65, 28.11 | 0.51 |
| Last meal | 518 | 30.22 (16.48) | 518 | 35.38 (20.33) | 518 | -5.16**** | -40.94, 30.62 | 0.53 |
| First snack | 411 | 12.74 (10.57) | 500 | 13.18 (11.17) | 407 | -0.24 | -26.13, 25.65 | 0.27 |
| Last snack | 411 | 13.85 (12.96) | 500 | 14.84 (13.71) | 407 | -0.97 | -27.25, 25.31 | 0.30 |
| Breakfast | 416 | 17.93 (7.91) | 482 | 18.40 (8.13) | 412 | -0.67 | -16.94, 15.60 | 0.53 |
| Lunch | 498 | 22.96 (10.92) | 516 | 25.82 (12.62) | 497 | -2.80**** | -28.03, 22.43 | 0.44 |
| Dinner | 515 | 30.04 (15.89) | 516 | 34.95 (18.96) | 514 | -4.95**** | -38.25, 28.35 | 0.52 |
| Eating window |  |  |  |  |  |  |  |  |
| Duration of eating window 1 (hours; decimal)^f^ | 518 | 10.68 (2.52) | 518 | 10.67 (1.88) | 518 | 0.01 | -4.09, 4.11 | 0.57 |
| Duration of eating window 2 (hours; decimal)^g^ | 518 | 10.99 (2.47) | 518 | 11.01 (1.89) | 518 | -0.02 | -4.11, 4.07 | 0.56 |
| Eating midpoint 1 (clock time; decimal)^f^ | 518 | 14.37 (1.37) | 518 | 14.23 (1.16) | 518 | 0.15*** | -1.62, 1.92 | 0.75 |
| Eating midpoint 2 (clock time; decimal)^g^ | 518 | 14.53 (1.38) | 518 | 14.40 (1.20) | 518 | 0.13** | -1.65, 1.91 | 0.75 |
| Time interval between sleep and eating (hours;  decimal) |  |  |  |  |  |  |  |  |
| Wake time and first eating occasion | 518 | 1.63 (1.62) | 518 | 1.67 (1.14) | 518 | -0.03 | -2.80, 2.74 | 0.53 |
| Wake time and first meal | 518 | 1.77 (1.84) | 518 | 1.79 (1.28) | 518 | -0.01 | -3.04, 3.02 | 0.57 |
| Last eating occasion and sleep time | 518 | 3.83 (1.60) | 518 | 4.19 (1.26) | 518 | -0.36**** | -3.52, 2.80 | 0.39 |
| Last meal and sleep time | 518 | 4.60 (1.38) | 518 | 4.62 (1.30) | 518 | -0.02 | -2.53, 2.49 | 0.56 |

CNBQ, Chrono-Nutrition Behavior Questionnaire; EMA, ecological momentary assessment; SD, standard deviation.

^a^ Meals included breakfast, lunch, and dinner. All time-related variables are shown in decimal format, with the unit of *clock time; decimal* (e.g., 23.80 means 11:48 PM, while 7.41 means 7:25 AM); *hours; decimal* (e.g., 7.56 means a duration of 7 h 34 min); or *minutes; decimal* (e.g., 19.78 means a duration of 19 min 47 sec).

^b^ Based on 2–9 days’ data (median 7 days).

^c^ Calculated as the CNBQ-based value minus the EMA food diary-based value (at the individual level). Paired comparison was made using the paired t-test: * P <0.05, ** P <0.01, *** P <0.001, and **** P <0.0001.

^d^ Calculated as mean difference plus-minus 1.96 SD of the difference.

^e^ All values were significant (P <0.0001).

^f^ Calculated using the start time of first eating occasion and the start time of last eating occasion.

^g^ Calculated using the start time of first eating occasion and the finish time of last eating occasion.

Supplemental Table 12. Sleep and temporal patterns of eating variables on non-workdays in younger participants (aged <44 years) ^a^

|  | CNBQ | | EMA food diaries ^b^ | | Paired | Mean | Limit of | Spearman |
| --- | --- | --- | --- | --- | --- | --- | --- | --- |
|  | n | Mean (SD) | n | Mean (SD) | n | difference ^c^ | Agreement ^d^ | correlation ^e^ |
| Sleep variables |  |  |  |  |  |  |  |  |
| Sleep time (clock time; decimal) | 514 | 23.99 (2.75) | 514 | 24.18 (1.74) | 514 | -0.20 | -5.74, 5.34 | 0.62 |
| Wake time (clock time; decimal) | 514 | 8.08 (1.64) | 514 | 7.92 (1.60) | 514 | 0.16** | -2.20, 2.52 | 0.73 |
| Daily sleep duration (hours; decimal) | 514 | 7.86 (1.40) | 514 | 7.40 (1.26) | 514 | 0.46**** | -2.35, 3.27 | 0.46 |
| Mid-sleep time (clock time; decimal) | 514 | 4.15 (1.36) | 514 | 4.26 (1.42) | 514 | -0.11** | -1.96, 1.74 | 0.78 |
| Daily eating frequency (number) |  |  |  |  |  |  |  |  |
| Meals | 514 | 2.63 (0.53) | 514 | 2.59 (0.45) | 514 | 0.04 | -0.79, 0.87 | 0.61 |
| Snacks | 514 | 1.29 (0.98) | 514 | 1.02 (0.82) | 514 | 0.27**** | -1.64, 2.18 | 0.45 |
| Total | 514 | 3.92 (1.17) | 514 | 3.61 (1.01) | 514 | 0.31**** | -1.85, 2.47 | 0.51 |
| Start time of eating (clock time; decimal) |  |  |  |  |  |  |  |  |
| First eating occasion | 514 | 9.74 (2.33) | 514 | 9.66 (2.08) | 514 | 0.08 | -3.04, 3.20 | 0.77 |
| Last eating occasion | 514 | 20.00 (1.65) | 514 | 19.72 (1.22) | 514 | 0.28**** | -2.74, 3.30 | 0.46 |
| First meal | 514 | 9.92 (2.54) | 514 | 9.81 (2.19) | 514 | 0.11 | -3.20, 3.42 | 0.78 |
| Last meal | 514 | 18.88 (0.90) | 514 | 19.09 (1.03) | 514 | -0.21**** | -2.07, 1.65 | 0.58 |
| First snack | 382 | 14.72 (3.62) | 460 | 15.07 (2.82) | 359 | -0.26 | -7.63, 7.11 | 0.30 |
| Last snack | 382 | 18.97 (3.26) | 460 | 17.41 (2.81) | 359 | 1.49**** | -5.45, 8.43 | 0.34 |
| Breakfast | 345 | 8.40 (1.14) | 450 | 8.56 (1.05) | 341 | 0.02 | -1.62, 1.66 | 0.70 |
| Lunch | 495 | 12.63 (0.81) | 506 | 12.79 (0.73) | 490 | -0.17**** | -1.75, 1.41 | 0.46 |
| Dinner | 512 | 18.90 (0.87) | 514 | 19.25 (0.89) | 512 | -0.35**** | -1.84, 1.14 | 0.62 |
| Duration of eating occasion (minutes; decimal) |  |  |  |  |  |  |  |  |
| First eating occasion | 514 | 18.33 (9.27) | 514 | 14.77 (7.11) | 514 | 3.56**** | -15.09, 22.21 | 0.44 |
| Last eating occasion | 514 | 19.40 (19.46) | 514 | 20.80 (12.39) | 514 | -1.40 | -43.34, 40.54 | 0.22 |
| First meal | 514 | 18.33 (9.27) | 514 | 19.09 (12.44) | 514 | -0.76 | -24.56, 23.04 | 0.57 |
| Last meal | 514 | 28.51 (16.33) | 514 | 34.13 (18.75) | 514 | -5.62**** | -39.73, 28.49 | 0.50 |
| First snack | 380 | 12.74 (10.93) | 460 | 15.67 (20.55) | 358 | -2.58** | -36.38, 31.22 | 0.35 |
| Last snack | 380 | 15.47 (17.27) | 460 | 18.33 (26.60) | 358 | -2.86** | -42.85, 37.13 | 0.41 |
| Breakfast | 355 | 16.12 (7.81) | 450 | 16.06 (7.79) | 347 | -0.19 | -14.46, 14.08 | 0.59 |
| Lunch | 497 | 22.07 (10.21) | 506 | 23.90 (11.57) | 491 | -1.92*** | -26.11, 22.27 | 0.46 |
| Dinner | 512 | 28.56 (16.34) | 514 | 33.96 (18.97) | 512 | -5.44**** | -40.22, 29.34 | 0.49 |
| Eating window |  |  |  |  |  |  |  |  |
| Duration of eating window 1 (hours; decimal)^f^ | 514 | 10.26 (2.65) | 514 | 10.06 (2.18) | 514 | 0.20* | -4.30, 4.70 | 0.55 |
| Duration of eating window 2 (hours; decimal)^g^ | 514 | 10.59 (2.60) | 514 | 10.41 (2.18) | 514 | 0.18 | -4.37, 4.73 | 0.53 |
| Eating midpoint 1 (clock time; decimal)^f^ | 514 | 14.87 (1.53) | 514 | 14.69 (1.31) | 514 | 0.18*** | -1.91, 2.27 | 0.75 |
| Eating midpoint 2 (clock time; decimal)^g^ | 514 | 15.03 (1.56) | 514 | 14.87 (1.34) | 514 | 0.17*** | -1.95, 2.29 | 0.75 |
| Time interval between sleep and eating (hours;  decimal) |  |  |  |  |  |  |  |  |
| Wake time and first eating occasion | 514 | 1.68 (1.81) | 514 | 1.77 (1.46) | 514 | -0.09 | -3.35, 3.17 | 0.53 |
| Wake time and first meal | 514 | 1.85 (1.99) | 514 | 1.90 (1.58) | 514 | -0.04 | -3.52, 3.44 | 0.58 |
| Last eating occasion and sleep time | 514 | 3.86 (1.77) | 514 | 4.33 (1.41) | 514 | -0.47**** | -3.97, 3.03 | 0.39 |
| Last meal and sleep time | 514 | 4.81 (1.59) | 514 | 4.71 (1.45) | 514 | 0.10 | -2.67, 2.87 | 0.59 |

CNBQ, Chrono-Nutrition Behavior Questionnaire; EMA, ecological momentary assessment; SD, standard deviation.

^a^ Meals included breakfast, lunch, and dinner. All time-related variables are shown in decimal format, with the unit of *clock time; decimal* (e.g., 23.99 means 11:59 PM, while 8.08 means 8:05 AM); *hours; decimal* (e.g., 7.86 means a duration of 7 h 52 min); or *minutes; decimal* (e.g., 18.33 means a duration of 18 min 20 sec).

^b^ Based on 2–9 days’ data (median 7 days).

^c^ Calculated as the CNBQ-based value minus the EMA food diary-based value (at the individual level). Paired comparison was made using the paired t-test: * P <0.05, ** P <0.01, *** P <0.001, and **** P <0.0001.

^d^ Calculated as mean difference plus-minus 1.96 SD of the difference.

^e^ All values were significant (P <0.0001).

^f^ Calculated using the start time of first eating occasion and the start time of last eating occasion.

^g^ Calculated using the start time of first eating occasion and the finish time of last eating occasion.

Supplemental Table 13. Sleep and temporal patterns of eating variables on non-workdays in older participants (aged ≥44 years) ^a^

|  | CNBQ | | EMA food diaries ^b^ | | Paired | Mean | Limit of | Spearman |
| --- | --- | --- | --- | --- | --- | --- | --- | --- |
|  | n | Mean (SD) | n | Mean (SD) | n | difference ^c^ | Agreement ^d^ | correlation ^e^ |
| Sleep variables |  |  |  |  |  |  |  |  |
| Sleep time (clock time; decimal) | 536 | 23.27 (2.41) | 536 | 23.70 (1.58) | 536 | -0.43**** | -5.32, 4.46 | 0.66 |
| Wake time (clock time; decimal) | 536 | 6.87 (1.30) | 536 | 6.83 (1.18) | 536 | 0.04 | -2.10, 2.18 | 0.70 |
| Daily sleep duration (hours; decimal) | 536 | 7.42 (1.40) | 536 | 6.95 (1.18) | 536 | 0.46**** | -2.19, 3.11 | 0.46 |
| Mid-sleep time (clock time; decimal) | 536 | 3.25 (1.66) | 536 | 3.39 (1.22) | 536 | -0.14* | -3.37, 3.09 | 0.73 |
| Daily eating frequency (number) |  |  |  |  |  |  |  |  |
| Meals | 536 | 2.81 (0.43) | 536 | 2.79 (0.36) | 536 | 0.02 | -0.68, 0.72 | 0.51 |
| Snacks | 536 | 1.23 (1.04) | 536 | 1.09 (0.82) | 536 | 0.14** | -1.82, 2.10 | 0.49 |
| Total | 536 | 4.04 (1.16) | 536 | 3.88 (0.95) | 536 | 0.16*** | -1.95, 2.27 | 0.52 |
| Start time of eating (clock time; decimal) |  |  |  |  |  |  |  |  |
| First eating occasion | 536 | 8.54 (1.90) | 536 | 8.49 (1.68) | 536 | 0.05 | -2.75, 2.85 | 0.70 |
| Last eating occasion | 536 | 19.62 (1.57) | 536 | 19.50 (1.15) | 536 | 0.12* | -2.60, 2.84 | 0.56 |
| First meal | 536 | 8.62 (2.06) | 536 | 8.60 (1.74) | 536 | 0.02 | -3.00, 3.04 | 0.71 |
| Last meal | 536 | 18.73 (1.12) | 536 | 18.87 (1.00) | 536 | -0.14** | -2.01, 1.73 | 0.64 |
| First snack | 370 | 14.41 (3.86) | 489 | 14.68 (3.06) | 367 | -0.21 | -7.86, 7.44 | 0.37 |
| Last snack | 370 | 18.63 (3.10) | 489 | 17.12 (2.75) | 367 | 1.36**** | -5.09, 7.81 | 0.37 |
| Breakfast | 458 | 7.93 (1.06) | 511 | 8.03 (0.98) | 455 | -0.02 | -1.76, 1.72 | 0.70 |
| Lunch | 521 | 12.48 (0.72) | 534 | 12.69 (0.72) | 520 | -0.20**** | -1.66, 1.26 | 0.52 |
| Dinner | 529 | 18.81 (0.90) | 534 | 19.03 (0.83) | 529 | -0.22**** | -1.53, 1.09 | 0.71 |
| Duration of eating occasion (minutes; decimal) |  |  |  |  |  |  |  |  |
| First eating occasion | 536 | 17.93 (11.35) | 536 | 15.31 (7.53) | 536 | 2.62**** | -20.55, 25.79 | 0.36 |
| Last eating occasion | 536 | 20.38 (21.53) | 536 | 20.43 (11.06) | 536 | -0.05 | -45.63, 45.53 | 0.19 |
| First meal | 536 | 17.93 (11.35) | 536 | 19.88 (13.47) | 536 | -1.95*** | -27.69, 23.79 | 0.52 |
| Last meal | 536 | 31.42 (20.64) | 536 | 37.94 (23.97) | 536 | -6.53**** | -43.34, 30.28 | 0.59 |
| First snack | 371 | 12.07 (11.10) | 489 | 13.08 (11.61) | 368 | -1.22 | -27.85, 25.41 | 0.30 |
| Last snack | 371 | 13.21 (13.39) | 489 | 14.43 (14.88) | 368 | -1.55 | -32.28, 29.18 | 0.38 |
| Breakfast | 459 | 16.56 (7.27) | 511 | 17.84 (9.06) | 456 | -1.62**** | -17.30, 14.06 | 0.56 |
| Lunch | 522 | 20.70 (10.34) | 534 | 23.85 (10.87) | 521 | -2.96**** | -22.98, 17.06 | 0.47 |
| Dinner | 531 | 31.32 (20.22) | 534 | 37.57 (22.78) | 530 | -6.31**** | -40.28, 27.66 | 0.60 |
| Eating window |  |  |  |  |  |  |  |  |
| Duration of eating window 1 (hours; decimal)^f^ | 536 | 11.07 (2.35) | 536 | 11.00 (1.83) | 536 | 0.07 | -4.00, 4.14 | 0.50 |
| Duration of eating window 2 (hours; decimal)^g^ | 536 | 11.41 (2.29) | 536 | 11.34 (1.85) | 536 | 0.07 | -4.03, 4.17 | 0.48 |
| Eating midpoint 1 (clock time; decimal)^f^ | 536 | 14.08 (1.28) | 536 | 14.00 (1.11) | 536 | 0.08* | -1.78, 1.94 | 0.69 |
| Eating midpoint 2 (clock time; decimal)^g^ | 536 | 14.25 (1.28) | 536 | 14.17 (1.14) | 536 | 0.08* | -1.81, 1.97 | 0.68 |
| Time interval between sleep and eating (hours;  decimal) |  |  |  |  |  |  |  |  |
| Wake time and first eating occasion | 536 | 1.68 (1.70) | 536 | 1.70 (1.36) | 536 | -0.02 | -2.72, 2.68 | 0.54 |
| Wake time and first meal | 536 | 1.75 (1.85) | 536 | 1.79 (1.47) | 536 | -0.04 | -2.97, 2.89 | 0.54 |
| Last eating occasion and sleep time | 536 | 3.48 (1.55) | 536 | 3.97 (1.25) | 536 | -0.49**** | -3.66, 2.68 | 0.38 |
| Last meal and sleep time | 536 | 4.16 (1.45) | 536 | 4.30 (1.34) | 536 | -0.14* | -2.82, 2.54 | 0.56 |

CNBQ, Chrono-Nutrition Behavior Questionnaire; EMA, ecological momentary assessment; SD, standard deviation.

^a^ Meals included breakfast, lunch, and dinner. All time-related variables are shown in decimal format, with the unit of *clock time; decimal* (e.g., 23.27 means 11:16 PM, while 7.46 means 7:28 AM); *hours; decimal* (e.g., 7.42 means a duration of 7 h 25 min); or *minutes; decimal* (e.g., 17.93 means a duration of 17 min 56 sec).

^b^ Based on 2–9 days’ data (median 7 days).

^c^ Calculated as the CNBQ-based value minus the EMA food diary-based value (at the individual level). Paired comparison was made using the paired t-test: * P <0.05, ** P <0.01, *** P <0.001, and **** P <0.0001.

^d^ Calculated as mean difference plus-minus 1.96 SD of the difference.

^e^ All values were significant (P <0.0001).

^f^ Calculated using the start time of first eating occasion and the start time of last eating occasion.

^g^ Calculated using the start time of first eating occasion and the finish time of last eating occasion.

Supplemental Table 14. Sleep and temporal patterns of eating variables on non-workdays in shift working participants ^a^

|  | CNBQ | | EMA food diaries ^b^ | | Paired | Mean | Limit of | Spearman |
| --- | --- | --- | --- | --- | --- | --- | --- | --- |
|  | n | Mean (SD) | n | Mean (SD) | n | difference ^c^ | Agreement ^d^ | correlation ^e^ |
| Sleep variables |  |  |  |  |  |  |  |  |
| Sleep time (clock time; decimal) | 246 | 23.97 (2.67) | 246 | 23.91 (2.24) | 246 | 0.06 | -6.09, 6.21 | 0.43 |
| Wake time (clock time; decimal) | 246 | 7.94 (1.85) | 246 | 7.86 (1.87) | 246 | 0.08 | -3.29, 3.45 | 0.62 |
| Daily sleep duration (hours; decimal) | 246 | 7.77 (1.65) | 246 | 7.16 (1.38) | 246 | 0.61**** | -3.12, 4.34 | 0.25 |
| Mid-sleep time (clock time; decimal) | 246 | 4.15 (1.93) | 246 | 4.32 (1.72) | 246 | -0.16 | -3.72, 3.40 | 0.67 |
| Daily eating frequency (number) |  |  |  |  |  |  |  |  |
| Meals | 246 | 2.59 (0.53) | 246 | 2.52 (0.47) | 246 | 0.07* | -0.75, 0.89 | 0.64 |
| Snacks | 246 | 1.30 (1.05) | 246 | 0.92 (0.78) | 246 | 0.38**** | -1.63, 2.39 | 0.45 |
| Total | 246 | 3.89 (1.24) | 246 | 3.44 (1.01) | 246 | 0.45**** | -1.78, 2.68 | 0.55 |
| Start time of eating (clock time; decimal) |  |  |  |  |  |  |  |  |
| First eating occasion | 246 | 9.75 (2.40) | 246 | 9.80 (2.23) | 246 | -0.05 | -3.19, 3.09 | 0.76 |
| Last eating occasion | 246 | 20.08 (1.73) | 246 | 19.77 (1.33) | 246 | 0.31** | -2.76, 3.38 | 0.41 |
| First meal | 246 | 9.94 (2.63) | 246 | 9.99 (2.33) | 246 | -0.04 | -3.46, 3.38 | 0.76 |
| Last meal | 246 | 19.06 (1.16) | 246 | 19.06 (1.16) | 246 | -0.01 | -2.07, 2.05 | 0.54 |
| First snack | 176 | 14.30 (3.70) | 210 | 15.42 (3.06) | 165 | -1.09*** | -8.28, 6.10 | 0.30 |
| Last snack | 176 | 19.06 (3.46) | 210 | 17.85 (2.70) | 165 | 1.36**** | -6.31, 9.03 | 0.23 |
| Breakfast | 156 | 8.22 (1.18) | 211 | 8.58 (1.15) | 154 | -0.15* | -1.98, 1.68 | 0.67 |
| Lunch | 237 | 12.64 (0.83) | 241 | 12.93 (0.87) | 234 | -0.28**** | -2.10, 1.54 | 0.45 |
| Dinner | 244 | 19.10 (1.03) | 245 | 19.34 (0.96) | 244 | -0.24**** | -1.98, 1.50 | 0.60 |
| Duration of eating occasion (minutes; decimal) |  |  |  |  |  |  |  |  |
| First eating occasion | 246 | 19.84 (14.31) | 246 | 15.70 (7.72) | 246 | 4.13**** | -22.41, 30.67 | 0.45 |
| Last eating occasion | 246 | 20.98 (20.64) | 246 | 22.34 (10.86) | 246 | -1.35 | -44.56, 41.86 | 0.25 |
| First meal | 246 | 19.84 (14.31) | 246 | 21.36 (12.51) | 246 | -1.52 | -28.59, 25.55 | 0.59 |
| Last meal | 246 | 30.44 (19.88) | 246 | 36.98 (19.33) | 246 | -6.54**** | -40.54, 27.46 | 0.50 |
| First snack | 176 | 12.68 (9.40) | 210 | 17.64 (16.42) | 165 | -4.28*** | -34.47, 25.91 | 0.34 |
| Last snack | 176 | 14.88 (11.95) | 210 | 18.18 (17.80) | 165 | -3.22* | -38.45, 32.01 | 0.40 |
| Breakfast | 158 | 16.27 (7.72) | 211 | 18.13 (9.09) | 156 | -1.92** | -17.83, 13.99 | 0.58 |
| Lunch | 239 | 21.87 (12.44) | 241 | 24.82 (12.24) | 235 | -2.79*** | -24.47, 18.89 | 0.49 |
| Dinner | 243 | 30.18 (18.87) | 245 | 36.79 (19.40) | 243 | -6.73**** | -40.56, 27.10 | 0.49 |
| Eating window |  |  |  |  |  |  |  |  |
| Duration of eating window 1 (hours; decimal)^f^ | 246 | 10.33 (2.80) | 246 | 9.97 (2.37) | 246 | 0.37* | -4.43, 5.17 | 0.57 |
| Duration of eating window 2 (hours; decimal)^g^ | 246 | 10.68 (2.71) | 246 | 10.34 (2.39) | 246 | 0.34* | -4.50, 5.18 | 0.55 |
| Eating midpoint 1 (clock time; decimal)^f^ | 246 | 14.92 (1.56) | 246 | 14.78 (1.40) | 246 | 0.13* | -1.84, 2.10 | 0.75 |
| Eating midpoint 2 (clock time; decimal)^g^ | 246 | 15.09 (1.57) | 246 | 14.97 (1.43) | 246 | 0.12 | -1.88, 2.12 | 0.75 |
| Time interval between sleep and eating (hours;  decimal) |  |  |  |  |  |  |  |  |
| Wake time and first eating occasion | 246 | 1.84 (1.88) | 246 | 2.01 (1.58) | 246 | -0.17 | -3.25, 2.91 | 0.56 |
| Wake time and first meal | 246 | 2.03 (2.09) | 246 | 2.18 (1.71) | 246 | -0.14 | -3.66, 3.38 | 0.58 |
| Last eating occasion and sleep time | 246 | 3.71 (1.87) | 246 | 4.21 (1.62) | 246 | -0.50**** | -4.34, 3.34 | 0.36 |
| Last meal and sleep time | 246 | 4.57 (1.72) | 246 | 4.64 (1.66) | 246 | -0.07 | -3.41, 3.27 | 0.47 |

CNBQ, Chrono-Nutrition Behavior Questionnaire; EMA, ecological momentary assessment; SD, standard deviation.

^a^ Meals included breakfast, lunch, and dinner. All time-related variables are shown in decimal format, with the unit of *clock time; decimal* (e.g., 23.97 means 11:58 PM, while 7.94 means 7:56 AM); *hours; decimal* (e.g., 7.77 means a duration of 7 h 46 min); or *minutes; decimal* (e.g., 19.84 means a duration of 19 min 50 sec).

^b^ Based on 2–9 days’ data (median 7 days).

^c^ Calculated as the CNBQ-based value minus the EMA food diary-based value (at the individual level). Paired comparison was made using the paired t-test: * P <0.05, ** P <0.01, *** P <0.001, and **** P <0.0001.

^d^ Calculated as mean difference plus-minus 1.96 SD of the difference.

^e^ All values were significant: P = 0.0001 for start time of first snack, 0.003 for start time of last snack, and <0.0001 for all other variables.

^f^ Calculated using the start time of first eating occasion and the start time of last eating occasion.

^g^ Calculated using the start time of first eating occasion and the finish time of last eating occasion.

Supplemental Table 15. Sleep and temporal patterns of eating variables on non-workdays in non-shift working participants ^a^

|  | CNBQ | | EMA food diaries ^b^ | | Paired | Mean | Limit of | Spearman |
| --- | --- | --- | --- | --- | --- | --- | --- | --- |
|  | n | Mean (SD) | n | Mean (SD) | n | difference ^c^ | Agreement ^d^ | correlation ^e^ |
| Sleep variables |  |  |  |  |  |  |  |  |
| Sleep time (clock time; decimal) | 804 | 23.51 (2.58) | 804 | 23.94 (1.46) | 804 | -0.43**** | -5.31, 4.45 | 0.73 |
| Wake time (clock time; decimal) | 804 | 7.31 (1.48) | 804 | 7.21 (1.33) | 804 | 0.10** | -1.68, 1.88 | 0.78 |
| Daily sleep duration (hours; decimal) | 804 | 7.59 (1.34) | 804 | 7.17 (1.19) | 804 | 0.42**** | -1.91, 2.75 | 0.55 |
| Mid-sleep time (clock time; decimal) | 804 | 3.55 (1.44) | 804 | 3.66 (1.24) | 804 | -0.12** | -2.42, 2.18 | 0.80 |
| Daily eating frequency (number) |  |  |  |  |  |  |  |  |
| Meals | 804 | 2.76 (0.46) | 804 | 2.75 (0.39) | 804 | 0.02 | -0.73, 0.77 | 0.55 |
| Snacks | 804 | 1.25 (1.00) | 804 | 1.10 (0.83) | 804 | 0.15**** | -1.75, 2.05 | 0.48 |
| Total | 804 | 4.01 (1.14) | 804 | 3.84 (0.97) | 804 | 0.17**** | -1.92, 2.26 | 0.51 |
| Start time of eating (clock time; decimal) |  |  |  |  |  |  |  |  |
| First eating occasion | 804 | 8.94 (2.10) | 804 | 8.84 (1.83) | 804 | 0.10 | -2.80, 3.00 | 0.75 |
| Last eating occasion | 804 | 19.72 (1.58) | 804 | 19.56 (1.13) | 804 | 0.16** | -2.65, 2.97 | 0.55 |
| First meal | 804 | 9.04 (2.28) | 804 | 8.95 (1.91) | 804 | 0.09 | -3.00, 3.18 | 0.76 |
| Last meal | 804 | 18.73 (0.96) | 804 | 18.95 (0.97) | 804 | -0.22**** | -2.01, 1.57 | 0.64 |
| First snack | 576 | 14.65 (3.75) | 739 | 14.71 (2.90) | 561 | 0.02 | -7.51, 7.55 | 0.37 |
| Last snack | 576 | 18.72 (3.09) | 739 | 17.10 (2.78) | 561 | 1.45**** | -4.93, 7.83 | 0.39 |
| Breakfast | 647 | 8.11 (1.11) | 750 | 8.20 (1.00) | 642 | 0.03 | -1.62, 1.68 | 0.72 |
| Lunch | 779 | 12.53 (0.75) | 799 | 12.68 (0.67) | 776 | -0.16**** | -1.58, 1.26 | 0.50 |
| Dinner | 797 | 18.78 (0.83) | 803 | 19.08 (0.82) | 797 | -0.30**** | -1.59, 0.99 | 0.68 |
| Duration of eating occasion (minutes; decimal) |  |  |  |  |  |  |  |  |
| First eating occasion | 804 | 17.60 (8.79) | 804 | 14.85 (7.19) | 804 | 2.76**** | -16.33, 21.85 | 0.38 |
| Last eating occasion | 804 | 19.57 (20.51) | 804 | 20.08 (11.94) | 804 | -0.51 | -44.56, 43.54 | 0.18 |
| First meal | 804 | 17.60 (8.79) | 804 | 18.92 (13.07) | 804 | -1.32** | -25.43, 22.79 | 0.52 |
| Last meal | 804 | 29.86 (18.34) | 804 | 35.80 (22.31) | 804 | -5.94**** | -41.91, 30.03 | 0.57 |
| First snack | 575 | 12.33 (11.47) | 739 | 13.40 (16.54) | 561 | -1.19 | -31.52, 29.14 | 0.32 |
| Last snack | 575 | 14.19 (16.45) | 739 | 15.79 (22.37) | 561 | -1.89* | -37.60, 33.82 | 0.39 |
| Breakfast | 656 | 16.40 (7.46) | 750 | 16.69 (8.35) | 647 | -0.78** | -15.71, 14.15 | 0.57 |
| Lunch | 780 | 21.21 (9.55) | 799 | 23.59 (10.88) | 777 | -2.35**** | -24.65, 19.95 | 0.46 |
| Dinner | 800 | 29.90 (18.35) | 803 | 35.50 (21.55) | 799 | -5.62**** | -40.15, 28.91 | 0.57 |
| Eating window |  |  |  |  |  |  |  |  |
| Duration of eating window 1 (hours; decimal)^f^ | 804 | 10.78 (2.44) | 804 | 10.72 (1.92) | 804 | 0.06 | -4.05, 4.17 | 0.53 |
| Duration of eating window 2 (hours; decimal)^g^ | 804 | 11.11 (2.40) | 804 | 11.05 (1.93) | 804 | 0.06 | -4.09, 4.21 | 0.51 |
| Eating midpoint 1 (clock time; decimal)^f^ | 804 | 14.33 (1.40) | 804 | 14.20 (1.18) | 804 | 0.13*** | -1.85, 2.11 | 0.73 |
| Eating midpoint 2 (clock time; decimal)^g^ | 804 | 14.49 (1.42) | 804 | 14.37 (1.21) | 804 | 0.13*** | -1.88, 2.14 | 0.73 |
| Time interval between sleep and eating (hours;  decimal) |  |  |  |  |  |  |  |  |
| Wake time and first eating occasion | 804 | 1.63 (1.71) | 804 | 1.64 (1.34) | 804 | -0.01 | -2.96, 2.94 | 0.53 |
| Wake time and first meal | 804 | 1.73 (1.86) | 804 | 1.74 (1.45) | 804 | -0.01 | -3.12, 3.10 | 0.56 |
| Last eating occasion and sleep time | 804 | 3.65 (1.60) | 804 | 4.13 (1.25) | 804 | -0.48**** | -3.64, 2.68 | 0.41 |
| Last meal and sleep time | 804 | 4.45 (1.50) | 804 | 4.46 (1.32) | 804 | -0.01 | -2.53, 2.51 | 0.63 |

CNBQ, Chrono-Nutrition Behavior Questionnaire; EMA, ecological momentary assessment; SD, standard deviation.

^a^ Meals included breakfast, lunch, and dinner. All time-related variables are shown in decimal format, with the unit of *clock time; decimal* (e.g., 23.51 means 11:31 PM, while 7.31 means 7:19 AM); *hours; decimal* (e.g., 7.59 means a duration of 7 h 35 min); or *minutes; decimal* (e.g., 17.60 means a duration of 17 min 36 sec).

^b^ Based on 2–9 days’ data (median 7 days).

^c^ Calculated as the CNBQ-based value minus the EMA food diary-based value (at the individual level). Paired comparison was made using the paired t-test: * P <0.05, ** P <0.01, *** P <0.001, and **** P <0.0001.

^d^ Calculated as mean difference plus-minus 1.96 SD of the difference.

^e^ All values were significant (P <0.0001).

^f^ Calculated using the start time of first eating occasion and the start time of last eating occasion.

^g^ Calculated using the start time of first eating occasion and the finish time of last eating occasion.

Supplemental Table 16. Social and eating jetlag variables and chronotype in each of subgroups ^a^

|  | CNBQ | | EMA food diaries ^b^ | | Paired | Mean | Limit of | Spearman |
| --- | --- | --- | --- | --- | --- | --- | --- | --- |
|  | n | Mean (SD) | n | Mean (SD) | n | difference ^c^ | Agreement ^d^ | correlation ^e^ |
| Male participants |  |  |  |  |  |  |  |  |
| Social jetlag (hours; decimal) | 532 | 0.94 (1.72) | 532 | 1.04 (1.33) | 532 | -0.09 | -2.78, 2.59 | 0.43 |
| Eating jetlag based on eating midpoint 1 (hours;  decimal) ^f^ | 532 | 0.95 (1.09) | 532 | 0.83 (0.79) | 532 | 0.12** | -1.90, 2.13 | 0.33 |
| Eating jetlag based on breakfast timing (hours;  decimal) | 366 | 1.19 (1.07) | 445 | 1.19 (0.94) | 363 | 0.08 | -1.99, 2.15 | 0.51 |
| Eating jetlag based on lunch timing (hours;  decimal) | 506 | 0.62 (0.76) | 520 | 0.62 (0.56) | 502 | 0.01 | -1.38, 1.40 | 0.33 |
| Eating jetlag based on dinner timing (hours;  decimal) | 523 | 0.90 (0.99) | 532 | 0.86 (0.80) | 523 | 0.04 | -1.66, 1.73 | 0.42 |
| Chronotype (clock time; decimal) | 532 | 3.35 (1.85) | 532 | 3.60 (1.46) | 532 | -0.25** | -3.75, 3.25 | 0.69 |
| Chronotype (clock time; decimal) among non-  users of clock alarm on non-workdays | 361 | 3.31 (1.38) | 361 | 3.66 (1.48) | 361 | -0.35**** | -2.74, 2.04 | 0.69 |
| Female participants |  |  |  |  |  |  |  |  |
| Social jetlag (hours; decimal) | 518 | 0.90 (1.53) | 518 | 0.89 (1.00) | 518 | 0.01 | -2.58, 2.60 | 0.39 |
| Eating jetlag based on eating midpoint 1 (hours;  decimal) ^f^ | 518 | 0.84 (0.92) | 518 | 0.71 (0.67) | 518 | 0.13*** | -1.57, 1.84 | 0.35 |
| Eating jetlag based on breakfast timing (hours;  decimal) | 393 | 1.15 (1.02) | 471 | 1.08 (0.86) | 390 | 0.11** | -1.46, 1.68 | 0.62 |
| Eating jetlag based on lunch timing (hours;  decimal) | 488 | 0.73 (0.69) | 516 | 0.65 (0.61) | 487 | 0.09** | -1.34, 1.52 | 0.27 |
| Eating jetlag based on dinner timing (hours;  decimal) | 510 | 0.70 (0.75) | 516 | 0.67 (0.65) | 510 | 0.03 | -1.43, 1.50 | 0.36 |
| Chronotype (clock time; decimal) | 518 | 3.27 (1.15) | 518 | 3.46 (1.18) | 518 | -0.20**** | -1.85, 1.46 | 0.74 |
| Chronotype (clock time; decimal) among non-  users of clock alarm on non-workdays | 298 | 3.32 (1.14) | 298 | 3.51 (1.20) | 298 | -0.19*** | -1.91, 1.52 | 0.74 |
| Younger participants (aged <44 years) |  |  |  |  |  |  |  |  |
| Social jetlag (hours; decimal) | 514 | 1.02 (0.88) | 514 | 1.06 (0.92) | 514 | -0.04 | -1.90, 1.82 | 0.38 |
| Eating jetlag based on eating midpoint 1 (hours;  decimal) ^f^ | 514 | 1.04 (1.09) | 514 | 0.90 (0.79) | 514 | 0.15** | -1.87, 2.16 | 0.34 |
| Eating jetlag based on breakfast timing (hours;  decimal) | 320 | 1.34 (1.01) | 429 | 1.27 (0.96) | 317 | 0.14** | -1.65, 1.94 | 0.52 |
| Eating jetlag based on lunch timing (hours;  decimal) | 481 | 0.74 (0.68) | 504 | 0.70 (0.62) | 477 | 0.05 | -1.37, 1.48 | 0.26 |
| Eating jetlag based on dinner timing (hours;  decimal) | 508 | 0.81 (0.83) | 514 | 0.78 (0.70) | 508 | 0.03 | -1.55, 1.62 | 0.36 |
| Chronotype (clock time; decimal) | 514 | 3.72 (1.30) | 514 | 3.94 (1.36) | 514 | -0.22**** | -2.22, 1.79 | 0.73 |
| Chronotype (clock time; decimal) among non-  users of clock alarm on non-workdays | 330 | 3.79 (1.25) | 330 | 4.01 (1.40) | 330 | -0.22*** | -2.38, 1.94 | 0.70 |
| Older participants (aged ≥44 years) |  |  |  |  |  |  |  |  |
| Social jetlag (hours; decimal) | 536 | 0.83 (2.11) | 536 | 0.87 (1.38) | 536 | -0.04 | -3.25, 3.17 | 0.39 |
| Eating jetlag based on eating midpoint 1 (hours;  decimal) ^f^ | 536 | 0.75 (0.91) | 536 | 0.65 (0.65) | 536 | 0.10** | -1.61, 1.82 | 0.31 |
| Eating jetlag based on breakfast timing (hours;  decimal) | 439 | 1.05 (1.05) | 487 | 1.02 (0.84) | 436 | 0.06 | -1.79, 1.91 | 0.58 |
| Eating jetlag based on lunch timing (hours;  decimal) | 513 | 0.61 (0.76) | 532 | 0.57 (0.54) | 512 | 0.04 | -1.36, 1.44 | 0.33 |
| Eating jetlag based on dinner timing (hours;  decimal) | 525 | 0.80 (0.94) | 534 | 0.75 (0.78) | 525 | 0.04 | -1.54, 1.62 | 0.42 |
| Chronotype (clock time; decimal) | 536 | 2.91 (1.65) | 536 | 3.14 (1.17) | 536 | -0.23** | -3.54, 3.08 | 0.65 |
| Chronotype (clock time; decimal) among non-  users of clock alarm on non-workdays | 329 | 2.84 (1.11) | 329 | 3.18 (1.18) | 329 | -0.34**** | -2.41, 1.72 | 0.66 |
| Shift working participants |  |  |  |  |  |  |  |  |
| Social jetlag (hours; decimal) | 246 | 1.21 (2.08) | 246 | 1.28 (1.39) | 246 | -0.06 | -3.73, 3.60 | 0.27 |
| Eating jetlag based on eating midpoint 1 (hours;  decimal) ^f^ | 246 | 1.20 (1.23) | 246 | 0.92 (0.82) | 246 | 0.28*** | -1.94, 2.51 | 0.31 |
| Eating jetlag based on breakfast timing (hours;  decimal) | 141 | 1.25 (1.16) | 201 | 1.17 (0.88) | 140 | 0.13 | -2.34, 2.61 | 0.26 |
| Eating jetlag based on lunch timing (hours;  decimal) | 228 | 0.85 (0.85) | 241 | 0.79 (0.63) | 227 | 0.08 | -1.55, 1.71 | 0.23 |
| Eating jetlag based on dinner timing (hours;  decimal) | 240 | 0.96 (1.05) | 245 | 0.96 (0.87) | 240 | 0.00 | -2.01, 2.01 | 0.27 |
| Chronotype (clock time; decimal) | 246 | 3.70 (1.90) | 246 | 3.96 (1.71) | 246 | -0.26* | -4.03, 3.52 | 0.58 |
| Chronotype (clock time; decimal) among non-  users of clock alarm on non-workdays | 152 | 3.77 (1.39) | 152 | 4.20 (1.84) | 152 | -0.43** | -3.59, 2.73 | 0.55 |
| Non-shift working participants |  |  |  |  |  |  |  |  |
| Social jetlag (hours; decimal) | 804 | 0.83 (1.45) | 804 | 0.87 (1.09) | 804 | -0.03 | -2.27, 2.20 | 0.46 |
| Eating jetlag based on eating midpoint 1 (hours;  decimal) ^f^ | 804 | 0.80 (0.92) | 804 | 0.73 (0.70) | 804 | 0.08* | -1.66, 1.81 | 0.34 |
| Eating jetlag based on breakfast timing (hours;  decimal) | 618 | 1.15 (1.01) | 715 | 1.13 (0.91) | 613 | 0.09** | -1.56, 1.73 | 0.65 |
| Eating jetlag based on lunch timing (hours;  decimal) | 766 | 0.62 (0.68) | 795 | 0.59 (0.56) | 762 | 0.04 | -1.30, 1.38 | 0.30 |
| Eating jetlag based on dinner timing (hours;  decimal) | 793 | 0.76 (0.83) | 803 | 0.71 (0.68) | 793 | 0.05 | -1.38, 1.48 | 0.42 |
| Chronotype (clock time; decimal) | 804 | 3.19 (1.39) | 804 | 3.40 (1.16) | 804 | -0.21**** | -2.56, 2.13 | 0.76 |
| Chronotype (clock time; decimal) among non-  users of clock alarm on non-workdays | 507 | 3.18 (1.21) | 507 | 3.41 (1.11) | 507 | -0.23**** | -1.91, 1.44 | 0.75 |

CNBQ, Chrono-Nutrition Behavior Questionnaire; EMA, ecological momentary assessment; SD, standard deviation.

^a^ Meals included breakfast, lunch, and dinner. All variables are shown in decimal format, with the unit of *hours; decimal* (e.g., 0.94 means a duration of 0 h 56 min) or *clock time; decimal* (e.g., 3.35 means 3:21 AM).

^b^ Based on 11 days’ data: 2–9 workdays’ data (median 7 days) and 2–9 non-workdays’ data (median 4 days).

^c^ Calculated as the CNBQ-based value minus the EMA food diary-based value (at the individual level). Paired comparison was made using the paired t-test: * P <0.05, ** P <0.01, *** P <0.001, and **** P <0.0001.

^d^ Calculated as mean difference plus-minus 1.96 SD of the difference.

^e^ All values were significant (P <0.0001).

^f^ Calculated using the start time of first eating occasion and the start time of last eating occasion.

^g^ Calculated using the start time of first eating occasion and the finish time of last eating occasion.
